# Biosynthetic and genetic pathways related to sialic acid metabolism

**DOI:** 10.1016/j.jbc.2026.111262

**Published:** 2026-02-06

**Authors:** Sjanie Huang, Eline G.P. van de Ven, Trisha Tee, Dirk J. Lefeber

**Affiliations:** 1 Department of Neurology, Donders Institute for Brain, Cognition and Behaviour, Radboud University Medical Center, Nijmegen, The Netherlands; 2 Department of Human Genetics, Radboud University Medical Center, Nijmegen, The Netherlands

**Keywords:** sialic acid, metabolism, congenital disorders of glycosylation, brain, platelet, skeletal muscle, human genetics, inherited metabolic disease, disease models, preclinical therapeutics

## Abstract

Sialic acid (Sia) is essential for human physiology and health, as emphasized by the range of human diseases that is linked to abnormalities in the Sia pathway. Sias are typically found at the outermost part of glycoconjugates that are involved in several biological processes, including cell adhesion and signaling. Sia metabolism is key to the production of cytidine 5′-monophosphate–Sia, the building block for sialylation, and is targeted as a therapeutic strategy to ameliorate the effects of abnormal sialylation in disease. Interestingly, patients with different genetic defects in Sia metabolism show contrasting clinical symptoms affecting different tissues. For example, neurological symptoms are dominant in some congenital disorders of glycosylation, like patients with a deficiency in *N*-acetylneuraminate synthase, causing NANS-CDG, while the brain is unaffected in patients with a deficiency in UDP-*N*-acetylglucosamine 2-epimerase/*N*-acetylmannosamine kinase which results in isolated muscle symptoms. This suggests that more complex tissue-specific regulatory mechanisms may exist. In this review, we discuss the biosynthetic and genetic pathways in Sia metabolism with a specific focus on its role in brain, muscle, and platelets in health and genetic disease. Moreover, this review presents an overview of the clinical symptoms and genetic spectrum for each genetic disease. Overall, the molecular and biochemical profiles are not fully understood in these patients, and effective therapies are limited. Therefore, additional research should focus on unravelling metabolic mechanisms that could be targeted to develop novel therapeutic strategies.

Sialic acid (Sia) is a summarizing term for a family of nine-carbon monosaccharides, typically found on the outer termini of glycoconjugates such as glycoproteins or glycolipids. Sialylated glycoconjugates are important for numerous cell biological processes in human physiology (1). Polysialic acid (polySia) and sialylated glycolipids, also called gangliosides, are important for normal brain development and function *via* modulating processes as synapse formation and synaptic plasticity (2). In the immune system, sialoglycans modulate interactions between immune cells and their targets, the activation of the complement system, and immunoglobulin function (3). Such functions are mediated by acting as linkage-specific recognition sites for interacting proteins, an important subgroup being the Sia-binding immunoglobulin-like lectins (Siglecs) (4). The human Siglec family, consisting of 14 members, is predominantly found on immune cells, and many members have important immune modulatory functions with most of the Siglecs mediating immune suppression (5). Siglecs are also expressed in the nervous system (6). Siglec function is related to the cell type and the ligands they interact with. For instance, Siglec-9 is present on neutrophils, monocytes, natural killer cells, and B lymphocytes. Other Siglecs are expressed on preferred cell types, like Siglec-1 on macrophages or Siglec-15 on myeloid cells and osteoclasts (7), and some cell types, such as neutrophils, can express multiple Siglecs (8, 9, 10). Sia–Siglec interactions play a vital role in balancing the activating and inhibitory signals of immune cells (4).

Dysregulation of glycoconjugate sialylation and their binding to glycan-binding proteins, such as Siglecs, has been associated with various human diseases. In many types of human cancer, sialylation is upregulated thereby promoting tumor metastasis. Viral entry is often dependent on sialylation of host receptors, and several immune and autoimmune diseases have been correlated with altered sialylation (11, 12, 13). While *N*-acetylneuraminic acid (Neu5Ac) is the predominant form of Sia in humans, dietary incorporation of *N*-glycolylneuraminic acid (Neu5Gc) in human tissues, derived from animal products, has been associated with potential health risks (14). The resulting natural immune response can trigger a chronic inflammatory process that may contribute to the progression of several disease phenotypes, including neurodegenerative disorders and cancer (15, 16, 17). In addition, 2-keto-3-deoxy-D-*glycero*-D-*galacto*-nononic acid or deaminated neuraminic acid (KDN), an uncommon form of Sia, has been correlated with several forms of cancer such as human ovarian and throat cancers (18, 19, 20). Sia metabolism has become an attractive therapeutic target for a range of common disorders such as atherosclerosis and cancer (21, 22, 23, 24, 25), because of its ease of intervention. Dietary Sia has been shown to improve cognitive function in animal studies, while inhibition of Sia metabolism using synthetic analogs is pursued as therapeutic strategy for cancer (26).

A growing number of patients are being diagnosed with genetic defects linked to the Sia pathway (Table 1). Currently, pathogenic variants have been described in seven genes related to Sia metabolism, leading to nine different phenotypes. In 1991, sialuria was the first disease described (27), caused by pathogenic variants in *GNE*, which encodes for the first committed enzyme in Sia biosynthesis. Interestingly, subsequently identified genetic diseases in the same pathway presented with tissue-specific symptoms, depending on which gene was affected. For example, muscle symptoms are observed in patients with pathogenic variants in the *GNE* or *NPL* gene, while brain function is affected when genetic defects are present in *NANS*, *CMAS*, or *SLC35A1*. Furthermore, platelets are impacted in patients with pathogenic variants in *GNE*, *NANS*, or *SLC35A1* with variable severity. Interestingly, pathogenic variants in *GNE* located outside the allosteric site can lead to myopathy, thrombocytopenia, or a combination of both symptoms, while patients with *GNE* variants in the allosteric site present with impaired brain development.

**Table 1:** Overview of genetic defects related to Sia metabolism

Disease gene	Phenotype MIM number	Inheritance	Main symptoms	Tissues involved	Total number of cases to date
*GNE*	605820	Autosomal recessive	Distal myopathy with rimmed vacuoles	Muscle	>1000
	620757	Autosomal recessive	Thrombocytopenia	Platelets	53
	269921	Autosomal dominant	Intellectual disability	Brain	11
*NANS*	610442	Autosomal recessive	Intellectual disability Seizures Facial dysmorphisms Skeletal dysplasia Thrombocytopenia	Brain Bone Platelets	21
*CMAS*	-	Autosomal recessive	Intellectual disability Facial dysmorphisms Abnormal walking gesture	Brain Bone	4
*SLC35A1*	603585	Autosomal recessive	Intellectual disability Seizures Thrombocytopenia	Brain Platelets	6
*ST3GAL3*	615006	Autosomal recessive	Intellectual disability	Brain	25
*ST3GAL5*	609056	Autosomal recessive	Developmental stagnation Hypo- or hyperpigmented skin macules	Brain Skin	126
*NPL*	-	Autosomal recessive	Myopathy Dilated cardiomyopathy	Muscle Heart	2

Genetic disorders affecting Sia metabolism are part of a larger group of congenital disorders of glycosylation (CDG), >180 in total (28). Current screening of CDGs with deficient *N*-glycosylation involves detection of altered glycosylation of glycoproteins in blood. The most convenient and commonly screened glycoprotein is transferrin due to its simple glycosylation with two biantennary N-glycans; however, this test is limited to defects in N-glycosylation (29). Further development of diagnostic and treatment options are lacking because of a poor understanding of the biochemical and clinical phenotypes. For patients with defects in Sia metabolism, a better understanding of Sia metabolism is essential, especially in the context of the affected tissues and the failing Sia supplementation trials for UDP-*N*-acetylglucosamine 2-epimerase/*N*-acetylmannosamine kinase (GNE) myopathy and *N*-acetylneuraminate synthase (NANS)-CDG. Several animal studies have demonstrated that the distribution and abundance of Sia types is different across organs (30, 31, 32). To understand the connection between tissue-specific effects and the symptoms observed in patients with impacted Sia metabolism, a comprehensive overview of genetic defects and the resulting symptoms is required. In this review, we will first present a brief overview of the different types of Sias and their metabolic pathways in humans. Next, we will discuss the role of Sias and their genetic defects with respect to muscle, brain, and platelets in order to identify the missing knowledge gaps and discuss potential intervention strategies.

## Sias and their metabolism in humans

Over 50 naturally occurring forms of Sia have been described in different species, mostly derived *via* biosynthetic modification of Neu5Ac. Besides Neu5Ac, two other variants found in human tissues are Neu5Gc and KDN (33, 34). Neu5Ac and Neu5Gc are the dominant Sia types present on the surface of most primate cell types (35, 36), while Neu5Ac is most dominant in human cells and tissues and is regarded as the canonical form in humans. Although Neu5Ac and Neu5Gc are structurally similar, with the *N*-acetyl group replaced by an *N*-glycolyl group, the human body recognizes Neu5Gc as nonhuman once exposed to the cell surface. KDN was identified most recently in humans. It lacks an *N*-acetyl group at carbon atom C-5 and instead contains a hydroxyl group. It was first discovered in cortical alveolar polysialoglycoprotein of rainbow trout eggs in 1986 (37). Although glycan-incorporated KDN was initially thought to be absent in mammals, free KDN was found in red blood cells (38), and more recently, KDN has been detected on human glycoproteins (39, 40).

### Biosynthesis of Neu5Ac

**Figure 1:**
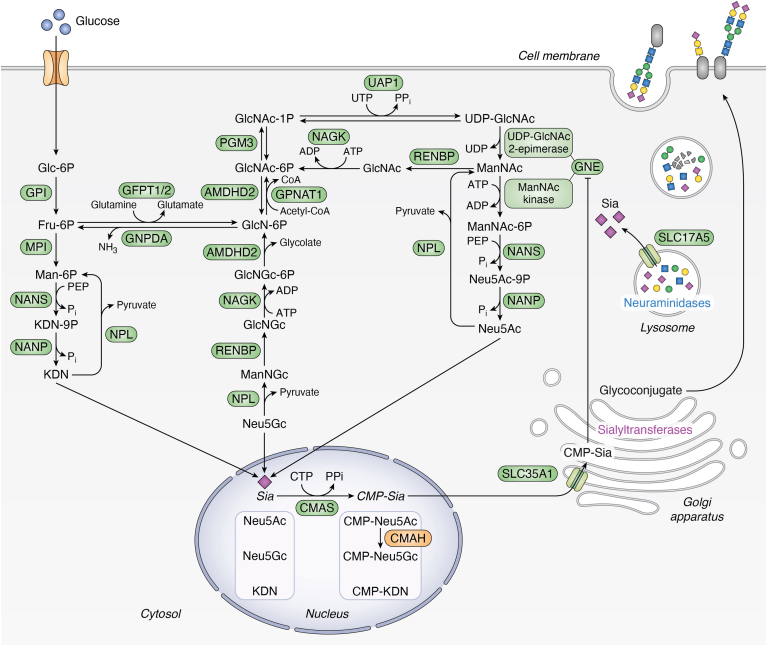
**Schematic overview of human sialic acid metabolism**. Sialic acid (Sia) *de novo* biosynthesis is mainly achieved in the cytosol, whereas its activation to CMP-Sia occurs in the nucleus. CMP-Sia is translocated to the Golgi apparatus, where attachment to newly synthesized glycoconjugates takes place by the action of sialyltransferases. Besides *de novo* synthesis, Sia can be recycled from glycans through the lysosomal salvage pathway; lysosomal neuraminidases can cleave Sia residues from endocytosed glycoconjugates that are transported into the cytosol *via* SLC17A5, enabling their reuse in cellular processes. This can involve reactivation to CMP-Sia by CMAS or catabolism by NPL, the function of which is currently unknown. As a sidenote, CMP-Neu5Ac can be used to synthesize CMP-Neu5Gc by the CMAH enzyme. However, due to an irreversible inactivation of the human *CMAH* gene, the human body can only obtain Neu5Gc through dietary uptake. Gene abbreviations: *GPI*, glucose 6-phosphate isomerase; *MPI*, mannose phosphate isomerase; *NANS*, *N*-acetylneuraminate synthase; *NANP*, *N*-acetylneuraminate 9-phosphate; *GFPT1/2*, glutamine-fructose 6-phosphate transaminase half; *GNPDA*, glucosamine 6-phosphate deaminase; *PGM3*, phosphoglucomutase 3; *AMDHD2*, amidohydrolase domain containing 2; *GPNAT1*, glucosamine phosphate *N*-acetyltransferase 1; NAGK, *N*-acetylglucosamine kinase; *RENBP*, renin binding protein; *NPL*, *N*-acetylneuraminate lyase; *UAP1*, UDP-*N*-acetylglucosamine pyrophosphorylase 1; *GNE*, UDP-*N*-acetylglucosamine 2-epimerase/*N*-acetylmannosamine kinase; *CMAS*, cytidine monophosphate *N*-acetylneuraminic acid synthetase; *CMAH*, cytidine monophospho-*N*-acetylneuraminic acid hydroxylase; *SLC35A1*, solute carrier family 35 member A1; *SLC17A5*, solute carrier family 17 member 5. Active human genes are displayed in *green*, and inactive human genes are displayed in *orange*. Metabolite abbreviations: Glc-6P, glucose 6-phosphate; Fru-6P, fructose 6-phosphate; Man-6P, mannose 6-phosphate; KDN-9P, deaminated neuraminic acid 9-phosphate; KDN, deaminated neuraminic acid; GlcNAc-1P, *N*-acetylglucosamine 1-phosphate; GlcNAc-6P, *N*-acetylglucosamine 6-phosphate; GlcN-6P, glucosamine 6-phosphate; GlcNGc, *N*-glycolylglucosamine; ManNGc, *N*-glycolylmannosamine; Neu5Gc, *N*-glycolylneuraminic acid; GlcNAc, *N*-acetylglucosamine; UDP-GlcNAc, uridine-diphospho-*N*-acetylglucosamine; ManNAc, *N*-acetylmannosamine; ManNAc-6P, *N*-acetylmannosamine 6-phosphate; Neu5Ac-9P, *N*-acetylneuraminic acid 9-phosphatase; Neu5Ac, *N*-acetylneuraminic acid; CMP, cytidine 5′-monophosphate; CoA, coenzyme A; ADP, adenosine diphosphate; ATP, adenosine triphosphate; PPi, inorganic pyrophosphate; UDP, uridine diphosphate; Pi, inorganic phosphate.

The biosynthesis of Neu5Ac in the cytosol is dependent on a carbon source, such as glucose, entering the cell (Fig. 1). Glucose is phosphorylated and converted in several enzymatic steps to fructose 6-phosphate, which can enter the hexosamine biosynthesis pathway. The product of the hexosamine biosynthesis pathway is the nucleotide sugar uridine diphosphate *N*-acetylglucosamine (UDP-GlcNAc), the initial substrate for nuclear production of cytidine 5′-monophosphate (CMP)-Neu5Ac, the nucleotide sugar precursor for protein sialylation. UDP-GlcNAc is first converted to *N*-acetylmannosamine (ManNAc) by the epimerase activity of the bifunctional enzyme GNE. Subsequently, the kinase activity of GNE is required to phosphorylate ManNAc to produce ManNAc 6-phosphate. Several studies suggest that *N*-acetylglucosamine kinase (NAGK) also holds ManNAc kinase activity (41, 42, 43). NAGK has been shown to play a role in the salvage pathway of GlcNAc for UDP-GlcNAc production, while its role in phosphorylation of ManNAc is not clear. NANS then converts ManNAc 6-phosphate to Neu5Ac 9-phosphate *via* addition of phosphoenolpyruvate, which is dephosphorylated by *N*-acetylneuraminic acid phosphatase (NANP) into Neu5Ac. Among the genes in the Sia biosynthesis pathway, only NANP has not been associated with any disease phenotype. A potential explanation is that NANP was shown to be nonessential for Neu5Ac production in human HAP1 cells, suggesting the existence of a redundant phosphatase involved in Neu5Ac synthesis (44). Such a redundant enzyme may be expressed in a cell-specific manner, resulting in very specific symptoms of a potential NANP-CDG. Neu5Ac is subsequently transported to the nucleus *via* unsolved mechanisms and is activated to CMP-Neu5Ac by cytidine monophosphate *N*-acetylneuraminic acid synthetase (CMAS) (45). Importantly, cytosolic CMP-Neu5Ac inhibits the epimerization reaction of GNE by binding to its allosteric site, providing negative feedback for Neu5Ac production (46). Finally, CMP-Neu5Ac is transported into the Golgi apparatus by the transmembrane transporter solute carrier family 35 member A1 (SLC35A1). It has been shown that SLC35A1 is also involved in α-dystroglycan O-mannosylation (47) and was later demonstrated to act as a dual nucleotide sugar transporter that can transport CDP-ribitol, which can also be transported into the Golgi apparatus by solute carrier family 35 member A4 (SLC35A4) (48).

### Incorporation of Sias in glycoconjugates by sialyltransferases

After transport of CMP-Sias into the Golgi apparatus, Sias are incorporated into newly synthesized glycoconjugates by sialyltransferases (Fig. 1). The abundance of Sias on cell surfaces has major biophysical effects and is tightly regulated by opposing actions of sialyltransferases and sialidases or neuraminidase (NEU) enzymes (49). Sialyltransferases are located as type II membrane proteins in the membrane of the Golgi apparatus and are responsible for transferring Sia from the donor substrate CMP-Sia to a carbohydrate acceptor (*e*.*g*.*,* oligosaccharides on proteins and lipids) (50). These enzymes are categorized into four categories based on the type of glycosidic linkage they form: β-galactoside α-2,3-sialyltransferases (ST3GALs), β-galactoside α-2,6-sialyltransferases (ST6GALs), α-*N*-acetylgalactosaminide α-2,6-sialyltransferases (ST6GalNAcs), and α-*N*-acetyl-neuraminide α-2,8-sialyltransferases (ST8Sias) (51). To date, 20 human sialyltransferases have been identified (1). While some sialyltransferases like ST6GAL1 are well characterized, many remain less well studied (52). Sialyltransferases can have shared, competing, or unique functions, making it difficult to dissect the molecular and biological functions of each individual sialyltransferase. Dysfunction of sialyltransferases ultimately leads to human disease, *e*.*g*.*,* described genetic variants for *ST3GAL3* and *ST3GAL5* (Table 1) (5). Additionally, sialyltransferases show tissue- and developmental stage-specific expression, emphasizing the importance of studying them in relevant physiological and glycan contexts (5).

### Recycling and catabolism of Sias

Sialidases or NEUs remove Sia from glycoconjugates and are found intracellularly, on the cell surface, and as free soluble mediators. Four types of neuraminidases (NEU1-4) have been identified in humans and classified by their intracellular locations and substrate specificities (51). NEU1 is primarily located in the lysosome and is involved in exocytosis, immune response, and phagocytosis through lysosomal cleavage of Sias from their corresponding glycoconjugates (53). NEU2 is mainly present in the cytosol and plasma membrane, participating in myoblast and neuronal differentiation. NEU3 and NEU4 are both involved in neuronal differentiation, apoptosis, and adhesion but operate from different locations. NEU3 is predominantly found on the plasma membrane, whereas NEU4 is located in lysosomes, mitochondria, and the endoplasmic reticulum (1). The diversity of their biological functions and substrate specificity mostly rely on their *in vitro* activity against three major classes of sialoglycoconjugates (54): glycolipids, glycoproteins, and free oligosaccharides (54). NEU1 primarily acts upon sialylated glycoproteins and oligosaccharides with lower activity against gangliosides, whereas NEU3 has a preference for gangliosides (55). NEU2 and NEU4 are active against all three types of sialylated glycoconjugates (56, 57). The ability of these enzymes to discriminate between the common Sia linkages (ɑ2,3-, ɑ2,6-, and ɑ2,8-) has been less systematically studied. All four NEUs are able to cleave both α2,3- and α2,6-linkages, with NEU2 having a preference for α2,3-linkages (53, 58). Additionally, NEU1, NEU3, and NEU4 also have activity against α2,8-linkages, although it is not as efficient (59). Recycling of Sia molecules is highly dependent on proper functioning of NEUs. Cytosolic Neu5Ac is mainly derived from the lysosomal salvage pathway (60), where it is cleaved from glycoconjugates by lysosomal NEU1 and transported into the cytoplasm *via* the lysosomal sialin transporter SLC17A5 (Fig. 1). Free Neu5Ac can be reused for sialylation either through conversion to CMP-Sia by CMAS to generate new glycoconjugates or through catabolism into ManNAc and pyruvate by the enzyme *N*-acetylneuraminate lyase (NPL) (61). In turn, ManNAc can re-enter the biosynthesis pathway for Neu5Ac or be further converted to GlcNAc by the renin-binding protein enzyme (62), possibly for further redirection to glycolysis, although this mechanism has not been confirmed in humans.

### Biosynthesis of Neu5Gc

Neu5Gc is generated by the hydroxylation of CMP-Neu5Ac to produce CMP-Neu5Gc *via* CMP-Neu5Ac hydroxylase (CMAH) (Fig. 1). (63). However, this *de novo* synthesis pathway is absent in humans due to a human-specific 92 base pair (bp) deletion in exon 6, which irreversibly inactivates the *CMAH* gene (64, 65). Despite this, Neu5Gc has been detected in several human tissues and can be taken up by micropinocytosis from exogenous dietary sources such as dairy and red meat (17, 66, 67). *In vitro* assays in human cells treated with exogenous Neu5Gc also showed extensive uptake and incorporation of Neu5Gc into cell surface glycans (66). Once inside the cell, Neu5Gc is released from Neu5Gc-glycoconjugates by lysosomal sialidases and translocated into the nucleus and can subsequently be activated by CMAS to form CMP-Neu5Gc (Fig. 1) (68). Finally, CMP-Neu5Gc is delivered to the Golgi apparatus where the Neu5Gc is attached to newly synthesized glycoconjugates (34, 66, 69, 70). Most ingested Neu5Gc is excreted *via* urine, with only a small portion being used for glycoconjugate synthesis (66). Neu5Gc can also be further metabolized by NPL to yield *N*-glycolylmannosamine and pyruvate (Fig. 1) (71, 72).

### Biosynthesis of KDN

Two pathways have been proposed for synthesis of KDN. The most scientifically supported pathway was first elucidated in trout testes where KDN was synthesized from mannose (73, 74). Mannose is phosphorylated to mannose 6-phosphate by hexokinase using ATP as a phosphate donor (Fig. 1). Subsequently, NANS converts mannose 6-phosphate to KDN 9-phosphate, which is likely dephosphorylated by NANP to yield KDN (75). A second, less probable mechanism is a direct conversion from Neu5Ac by deacetylation and deamination reactions, though no evidence has proven that this pathway exists in tissue homogenates so far (76). In some species, KDN can be converted to CMP-KDN by CMAS, but this reaction is not preferred in human cells, likely explaining why KDN is rarely found in human glycoconjugates (68, 77). The exact pathway of KDN catabolism in humans is still unknown.

## Disorders of Sia metabolism affecting the brain

Neurological symptoms are common among the genetic defects in Sia metabolism. This is not surprising, since out of all human tissues, Sia is most abundantly present in the brain (6, 78). During early postnatal years, Sia presence rapidly increases and can be influenced by dietary Sia intake to improve cognitive function, indicating its importance in early brain development (24). This was supported by preclinical studies from Mijdam *et al*. showing that inhibition of the Sia biosynthesis pathway leads to disturbed neural network formation in induced pluripotent stem cell (iPSC)–derived developing neurons (79). At approximately 50 years of age, Sia levels plateau and begin to gradually decline (80). While Sias in most tissues are mainly present on N- and O-linked glycoproteins, in the brain, they are predominantly incorporated into gangliosides. Gangliosides are complex glycolipids that are essential for a broad range of neuronal functions, including nerve cell excitability, axon–myelin interactions, and axon stability and regeneration (60, 61). Over 100 different ganglioside structures have been identified in the brain, with concentrations of ganglioside-bound Sias varying with age (81). Sias are also abundantly present on neural cell adhesion molecule (NCAM) in the form of polySia. NCAM is expressed on the surface of cells in the central nervous system (CNS), playing a key role in synapse formation, neuronal plasticity, and memory (80). PolySia is a unique homopolymer consisting of >90 α-2,8-linked Sia monomers that are formed by two polysialyltransferases, ST8Sia-II and ST8Sia-IV (82). PolySia is mainly detected in four cell types and acts upon several important developmental events, including migrating neuroblast cells, extending cells like neurons and Schwann cells, and neural stem cells. PolySia is also highly present in synapses and involved in synaptic plasticity (83, 84). More extensive information on gangliosides [accepted review] and brain function [accepted review] can be found in other Sia reviews.

The regulation of Sia in maintaining healthy brain function is highly dynamic and sensitive to internal and external cues. Rapid changes in sialylation on the cell surface can occur physiologically, often induced by the transfer of NEU1 or NEU4 to the cell surface (85). A mouse study showed that acute stress could induce a rapid decrease of polysialylation in the olfactory bulb and prefrontal cortex, mediated by sialidases secreted from microglia and astrocytes (86). Similarly, neuronal activity was shown to increase sialidase activity on the neuronal and astrocytic surface, causing neuronal desialylation and resulting in aberrant memory formation (87). These findings highlight how even transient disturbances in the regulation of membrane-bound Sias can have significant functional consequences. When this regulatory balance is chronically disrupted, such as through genetic mutations, the effects are often more severe and permanent. Genetic deficiency of sialyltransferases ST3GAL3 (OMIM#611090) and ST3GAL5 (OMIM#609056), for example, two enzymes that add Sia residues to glycoconjugates, leads to infantile epilepsy, developmental delay, and blindness (88, 89, 90, 91). Additionally, an ST3GAL2/3 double knockout (KO) mouse was presented with demyelination and a reduction in neuronal markers, leading to multiple neurological symptoms (92). Moreover, recently discovered congenital disorders such as NANS-CDG (OMIM#605202) and CMAS deficiency provide further evidence that an intact Sia metabolism itself is indispensable for normal brain development and function. The following sections will explore these disorders in Sia metabolism in more detail.

### NANS-CDG

#### Clinical symptoms

NANS is the enzyme converting ManNAc 6-phosphate to Neu5Ac 9-phosphate (Fig. 1). Genetic variants in *NANS* cause NANS-CDG (OMIM#605202), an autosomal recessive disorder first described in 2016 by van Karnebeek *et al*. (93). To date, 21 patients have been reported worldwide that are represented with a number of hallmark clinical features, including intellectual developmental disorder with a delay in developmental milestones, short stature with short limbs, neurological impairment, skeletal dysplasia, seizures, and facial dysmorphisms (93, 94, 95, 96). Additional clinical features observed in several patients in a cohort study by den Hollander *et al*. include gastrointestinal dysfunction, thrombocytopenia, ophthalmological abnormalities, an abnormal septum pellucidum, (progressive) cerebral atrophy, and ventricular dilatation (96). The most recent NANS-CDG case report published by Yoo *et al*. further expanded the phenotypic spectrum with endocrinopathy (97).

#### Genetic spectrum and molecular and biochemical profile

The *NANS* gene, located on chromosome 9, consists of six exons encoding a 1080-bp sequence, resulting in a 359 amino acid-protein that contains three domains: the N-terminal, the *N*-acetylneuraminic acid synthase domain, and the C-terminal antifreeze type III domain (96). The initial study by van Karnebeek *et al*. described the first 10 monoallelic genetic variants in *NANS*, of which six missense variants [c.85 C > A, p.(His29Asn); c.398 G > T, p.(Gly133Val); c.452 G > A, p.(Arg151His); c.562 T > C, p.(Tyr188His); c.566 C > T, p.(Pro189Leu); and c.709 C > T, p.(Arg237Cys)] and one triplet insertion variant (c.979_981dup, p.(Ile327dup). All seven variants resulted in a pathogenic effect on protein function, predicted by a constructed three-dimensional protein model for NANS (93). The missense mutations causing p.(Gly113Val), p.(Tyr188His), and p.(Pro189Leu) are close to the active site, and likely affect the catalytic activity of the NANS enzyme by disrupting substrate binding, pocket shape, or affecting functional residues. The p.(His29Asn) and p.(Arg151His) substitutions are located at the dimer interface and likely affect protein folding, stability, and dimerization. The p.(Ile327dup) variant potentially affects protein folding and lastly (p.Arg151His) is located at the protein surface, possibly interfering with folding or protein–protein interactions (93). The remaining three variants resulted either in reduced levels of the canonical *NANS* transcript or in alternatively spliced *NANS* transcripts that are degraded by nonsense-mediated decay (NMD). The indel variant (c.449–10_449-5delinsATGG, p.?) resulted in very low levels of mRNA of both the canonical *NANS* transcript and an isoform with in-frame exon 3 and 4 exclusion. Similarly, the splice-site variant c.448+1G > A (p.?) produced two splicing isoforms, one again lacking exons 3 and 4, expressed at levels equal to the canonical *NANS* transcript, and a second out-of-frame isoform with very low expression levels. The last variant found in this study was a single-nucleotide insertion c.389dup [p.(Lys131Glnfs∗8)] resulting in a transcript triggering NMD (93).

Den Hollander *et al*. expanded the genetic spectrum with eight novel monoallelic variants [c.1 A > G, p.(Met1?); c.88 C > T, p.(Gln30∗); c.92del, p.(Gly31Alafs∗5); c.200 T > G, p.(Leu67Trp); c.351 G > A, p.(Met117Ile); c.440 C > A, p.(Ala147Asp); c.710 G > A, p.(Arg237His); and c.922_925dup, p.(Met309Asnfs∗11)], all predicted to be highly pathogenic, though this was not further explored (96). Masunaga *et al*. further investigated three novel monoallelic variants in Japanese NANS-CDG patients (98). The first missense variant c.207del [p.(Arg6Serfs∗57)] was evaluated as pathogenic, the second intronic variant c.133-12T > A (p.(=)) created a novel splice acceptor site, producing an aberrant mRNA transcript undergoing NMD, and the last missense variant c.607 T > C (p.(Tyr203His)) caused reduced canonical *NANS* mRNA expression (99). Most recently, two newly identified biallelic variants of *NANS* in the first Korean patient were reported (97). These two missense variants c.735 G > A (p.(Trp245∗)) and c.668 T > C (p.(Il223Thr)) classified as likely pathogenic. To conclude, there are 23 monoallelic variants reported in *NANS* so far, of which 17 are located within the synthase domain, four affect the N-terminal, and two variants are located in the antifreeze type III domain. An overview of all *NANS* variants described is provided in Table 2.

**Table 2:** All genetic variants identified and documented in NANS-CDG patients

Nucleotide substitution NM_018946.4	Amino acid substitution NP_061819.2	*NANS* exon	NANS protein domain	Variant type	Clinical classification (ClinVar)	Reference
c.1 A > G	p.(Met1?)	1	N-terminal	Start loss	Likely pathogenic	(96)
c.85 C > A	p.(His29Asn)	1	N-terminal	Missense	Not classified	(93)
c.88 C > T	p.(Gln30∗)	1	N-terminal	Nonsense	Not classified	(96)
c.92del	p.(Gly31Alafs∗5)	1	N-terminal	Frameshift	Pathogenic	(96)
c.133–12T > A	p.(=)^a^	Intron 1	Synthase	Intron variant	Pathogenic	(99)
c.200 T > G	p.(Leu67Trp)	2	Synthase	Missense	Not classified	(96)
c.207del	p.(Arg6Serfs∗57)	2	Synthase	Frameshift	Likely pathogenic	(99)
c.351 G > A	p.(Met117IIe)	3	Synthase	Missense	Benign	(96)
c.389dup	p.(Lys131Glnfs∗8)	3	Synthase	Frameshift	Pathogenic	(93)
c.398 G > T	p.(Gly133Val)	3	Synthase	Missense	Pathogenic	(93)
c.440 C > A	p.(Ala147Asp)	3	Synthase	Missense	Not classified	(96)
c.448+1G > A	p.?	Intron 3	Synthase	Splice-altering variant	Pathogenic	(93)
c.449–10_449-5delinsATGG	p.?	Intron 3	Synthase	Splicing-altering variant	Pathogenic	(93, 96)
c.452 G > A	p.(Arg151His)	4	Synthase	Missense	VUS	(93)
c.562 T > C	p.(Tyr188His)	4	Synthase	Missense	Pathogenic	(93, 96)
c.566 C > T	p.(Pro189Leu)	4	Synthase	Missense	Not classified	(93)
c.607 T > C	p.(Tyr203His)	5	Synthase	Missense	Likely pathogenic	(99)
c.668 T > C	p.(Ile223Thr)	5	Synthase	Missense	VUS	(97)
c.709 C > T	p.(Arg237Cys)	5	Synthase	Missense	Pathogenic	(93, 96)
c.710 G > A	p.(Arg237His)	5	Synthase	Missense	Not classified	(96)
c.735 G > A	p.(Trp245∗)	5	Synthase	Nonsense	Likely pathogenic	(97)
c.922_925dup	p.(Met309Asnfs∗11)	6	Antifreeze type III	Frameshift	Not classified	(96)
c.979_981dup	p.(Ile327dup)	6	Antifreeze type III	In-frame insertion	VUS	(93, 99)

The location of the variant on the exon (or intron) number and on the protein domain are given. An asterisk (∗) indicates a premature stop codon. The clinical classification of the variants are reported based on the Clinical Variation (ClinVar) database.

a Production of aberrant mRNA degraded by NMD.

Biochemically, Sia incorporation in N-glycans of serum transferrin was normal, as well as Sia levels in urine (25). Interestingly, NANS-CDG patients showed elevated ManNAc levels in urine and plasma correlating significantly with clinical severity, providing a valuable diagnostic biomarker for the pathogenicity of *NANS* variants (94, 95, 96).

#### Disease models and therapeutic strategies

To gain more insight into the underlying disease mechanisms, several models have been established. A *nansa* knockdown (KD) in zebrafish resulted in embryos with a small head and skeletal abnormalities, a clinical feature often observed in NANS-CDG patients (93). A heterozygous *Nans*^+/−^ mouse model showed significant reduction of migrated neuronal cells in the ventricular zone, suggesting cortical neurogenesis impairment in the brain. These mice also showed neurobehavioral abnormalities like impaired contextual fear conditioning (100). However, while zebrafish, mice, and humans share key enzymatic processes of Sia metabolism, differences exist in the number of Sia variants produced, specific roles of certain enzymes, and their ability to synthesize certain Sia variants (64, 101, 102). To study more human-specific mechanisms, Bu *et al*. generated *NANS*-KO human cortical organoids using human iPSCs (100). These organoids lacked Sia and protein polysialylation, leading to reduced neural progenitor proliferation and expansion, dysregulated neural migration, impaired synapse formation, and neuronal hypoexcitability (100). While this model is human specific, it is important to note that a KO model results in a fully depleted enzyme, whereas NANS-CDG patients show residual enzyme activity (93).

Although currently no effective treatments are available for NANS-CDG patients, oral Neu5Ac supplementation could potentially be a promising therapeutic option. Similar sugar supplementation approaches have been effective in several other CDG subtypes, such as galactose supplementation in SLC35A2-CDG (OMIM#300896) (103) and fucose supplementation in SLC35C1-CDG (OMIM#605881) (104). Hypothetically, supplemented Neu5Ac could be used for intracellular formation of CMP-Neu5Ac (46). A study by Tran *et al*. in 2021 provided the first insights into the pharmacokinetics of orally administered Neu5Ac in NANS-CDG patients and healthy controls (105). Free Neu5Ac was rapidly absorbed but also quickly excreted in urine, with no significant impact on ManNAc levels in plasma and urine in either group (105). The lack of effect could suggest that oral Neu5Ac supplementation is not sufficient to increase Neu5Ac levels in plasma due to rapid urinary clearance (105) which would require adaptation of the pharmaceutical formulation or that cellular uptake of Neu5Ac is ineffective. Alternatively, since Sia levels in urine and on N-glycans appeared normal in NANS-CDG patients, it is possible that Sia may not be the only key player in the underlying disease mechanism. One final explanation could be that free Neu5Ac may have been partially hydrolyzed to ManNAc by NPL using the catabolic pathway instead of conversion to CMP-Sia. However, the latter seems less evident, since no effect on ManNAc levels was observed. A follow-up study in 2023 was performed in which both prenatal and postnatal Neu5Ac treatment was administered (106). Unfortunately, postnatally treated patients did not show significant biochemical or clinical improvement in any of the outcome measures, including cognitive tests, height and weight, seizure control, bone health, and gastrointestinal symptoms (106). The prenatally treated patient demonstrated improved development and social interaction postnatally compared to a patient with the same pathogenic variants, suggesting that the effect of Sia treatment may depend on timing. However, the evidence is limited due to small sample size, the lack of a proper control group, and the heterogeneity in genotype, phenotype, and age. While Neu5Ac supplementation as a treatment for NANS-CDG is still promising, long-term follow-up studies with larger cohorts, and possibly as different formulations, doses, or slower release of Sia in the system and studies on the efficiency of Sia uptake by different human tissues in general will be needed to verify these findings.

### GNE sialuria

#### Clinical symptoms and genetic spectrum

GNE is a bifunctional enzyme catalyzing the first two steps in Sia biosynthesis (Fig. 1). Enzyme function is inhibited through feedback inhibition by binding of CMP-Neu5Ac, the final product of this pathway, to the allosteric binding site of GNE (107). Genetic variations in this allosteric site affect CMP-Neu5Ac binding and therefore its feedback inhibition, resulting in excessive production of Sia and (French type) *GNE* sialuria (OMIM#269921) (108). Although the first patient was reported nearly 50 years ago (109, 110), its molecular characterization was not accomplished until 30 years later (108). To date, 11 sialuria patients have been described with heterozygous genetic variants in *GNE* causing sialuria in an autosomal dominant manner (108, 109, 110, 111, 112, 113, 114, 115, 116, 117). Three genetic variants have been detected and are described below (Table 3), all located in the allosteric binding site of the UDP-GlcNAc 2-epimerase domain (112), where CMP-Neu5Ac binds and initiates feedback inhibition (27, 108, 115).

**Table 3:** All genetic variants identified and documented in *GNE* sialuria patients

Nucleotide substitution NM_001128227.3	Amino acid substitution NP_001121699.1	*GNE* exon	GNE protein domain	Variant type	Clinical classification (ClinVar)	Reference
c.788 G > T	p.(Arg294Leu)	5	Epimerase	Missense	Likely pathogenic	(108, 109, 110, 111)
c.797 G > A	p.(Arg266Gln)	5	Epimerase	Missense	Pathogenic	(108, 112, 113, 114, 115, 116)
c.798 C > T	p.(Asn348 = )	5	Epimerase	Synonymous	Likely benign	(108, 117)

Clinically, excessive urinary excretion of free Sia is a metabolic hallmark of sialuria (113). Furthermore, patients present with (mild) neuromotor and cognitive developmental delay, fine motor deficits, seizures, hypotonia, slightly coarse facial features, and recurrent respiratory infections (108, 109, 110, 111, 112, 113, 114, 115, 116, 117). In some cases, children with sialuria also show delayed skeletal development with signs of dysostosis multiplex, but this is not a primary feature (115). Sialuria is challenging to recognize during infancy due to inconsistent and relatively subtle features, but measuring urinary free Sia offers a useful diagnostic marker in young children with mild developmental delay (112, 118).

#### Molecular and biochemical profile

Three isoforms are known for GNE: GNE1, GNE2, and GNE3. The universally expressed GNE1 is the original GNE protein of 722 amino acids and supports the main Sia supply (119). GNE2 has reduced UDP-GlcNAc 2-epimerase activity, and GNE3 possesses only kinase activity (120). GNE2 and GNE3 display tissue-specific patterns; kidney, liver, placenta, and colon express both GNE2 and GNE3, and GNE2 is additionally found in brain, lung, and pancreas (121).

In sialuria patients, all reported genetic variants affect the allosteric CMP-binding site of the UDP-GlcNAc 2-epimerase domain, resulting in normal GNE enzyme activity but impaired feedback inhibition (114). Sia levels were strongly elevated in urine of all cases. Patient-derived fibroblasts show significantly reduced suppression of epimerase activity when exposed to CMP-Neu5Ac compared to control fibroblasts (27). Increased CMP-Neu5Ac was consequently detected in patient fibroblasts. Serum protein examination additionally confirmed that apolipoprotein C-III (apoC-III), a glycoprotein carrying monosialylated and disialylated core 1 mucin O-glycans was hypersialylated, indicating that multiple glycosylation pathways are affected by increased CMP-Neu5Ac levels (122). Serum total O-glycans were also hypersialylated, making GNE sialuria the first metabolic disorder presenting with hypersialylated O-glycans. N-linked glycosylation is mainly unaffected (123).

#### Disease models and therapeutic strategies

The relation between the clinical phenotype and biochemical abnormalities is not well understood. Studies in Chinese hamster ovary (CHO) cells expressing the *GNE* sialuria variant c.788 G > T showed dramatically increased expression of polysialylated NCAM, which has been linked to neurodevelopmental delay (124). Similar results were found in a transgenic mouse expressing the c.788 G > T variant, highlighting that increased polySia on NCAM could play a role in the manifestation of the neurodevelopmental phenotype observed in sialuria patients (125).

Currently, the only treatment available for sialuria patients is symptomatic and supportive management, including seizure treatment and rehabilitation to optimize mobility (126). However, therapeutic treatment options based on sugar supplementation have been proposed. To decrease the observed intracellular Sia and increase CMP-Sia levels, sialuria fibroblasts were supplemented with exogenous cytidine triphosphate to enhance Sia to CMP-Sia conversion. Cytidine triphosphate feeding significantly decreased intracellular Sia and restored CMP-Sia-mediated feedback inhibition (111), making it a strong potential therapeutic candidate for sialuria (27). Furthermore, allele-specific gene silencing *via* small interfering RNAs is another potential therapeutic strategy in autosomal dominant diseases (127). Silencing of the mutant *GNE* allele in sialuria fibroblasts normalized free Sia levels and restored feedback inhibition of epimerase activity (127), suggesting feasibility for targeting dominant pathogenic alleles. Altogether, potential treatment approaches have been explored in *in vitro* models, yet additional research is required to evaluate their efficacy in humans.

### CMAS deficiency

#### Clinical symptoms and genetic spectrum

The CMAS enzyme activates Sia in the nucleus to CMP-Sia, an important step preceding its translocation to the Golgi by SLC35A1 (Fig. 1). Mechanisms that regulate CMAS are still relatively poorly characterized (128, 129). To date, a single *CMAS* homozygous variant (c.563 G > A) has been described in four individuals from the same family with an autosomal recessive disorder. Clinical features included congenital intellectual disability, speech impairment, and facial dysmorphisms resembling those of NANS-CDG patients and abnormal gait indicating possible skeletal involvement (129).

#### Molecular and biochemical profile

The CMAS protein localizes predominantly to the nucleus *via* basic cluster 2, a specific nuclear localization signal essential for both correct import to the nucleus and catalytic enzyme activity (130, 131). Unlike all other enzymes involved in Sia biosynthesis, the N-terminal CMAS sequence is well conserved across species, particularly in five structural motifs essential for enzymatic function (132, 133, 134). While the N domain of CMAS is highly conserved, the C domain only exists in vertebrates and some types of bacteria (133, 135, 136, 137). Wu *et al*. unraveled its importance in protein solubility using a medaka model with an L304Q point mutation in the C domain. This mutation resulted in increased free Sia, and when the L304Q variant was expressed in CHO cells, the protein accumulated in an insoluble fraction (138).

The CMAS variant p.Arg188His (c.563 G > A) was found in one of the strictly conserved motifs and predicted to influence protein dimerization, subsequently affecting protein activity (129). Despite altered structure, the CMAS variant showed greater substrate affinity and catalytic efficiency compared to wildtype CMAS. With increased CMAS activity, more CMP-Neu5Ac is synthesized, suggesting dysregulation rather than loss of function may impact disease pathology (129). Biochemical patient data to confirm elevated free Sia levels or altered glycan levels are still lacking.

#### Disease models and therapeutic strategies

*Drosophila* models have been instrumental in studying CMAS function, Csas in *Drosophila*, and its enzymatic domains. Islam *et al*. revealed that Csas is highly involved in neural transmission at neuromuscular junctions and in neuronal excitability in Csas KD and KO *Drosophila* flies (139). Interestingly, *Drosophila* Csas was found in the *trans*-Golgi, where sialylation occurs (139), in contrast to nuclear localization of all mammalian CMAS orthologs (130). Supporting this, Münster *et al*. showed that nuclear localization is not strictly required for CMAS function (131). A study by Scott *et al*. revealed that Csas is required in glial cells, whereas sialyltransferase is strictly present in neurons (140), highlighting that Csas is essential for regulating neuronal excitability. Even though ∼40% of genes are conserved between *Drosophila* and humans, there are significant limitations of using *Drosophila* to model human disease: they lack complex cognitive abilities, they do not have an adaptive immune system, and evident anatomical differences limit the study of specific tissues (141), while additionally Sia containing glycoconjugates are highly limited in this model system. *Cmas*-deficient mice die early during embryonic development (142), likely caused by a maternal complement attack against fetal trophoblast cells that lack sialoglycans which subsequently impairs extraembryonic tissue development (143). There are currently no records of potential therapeutic strategies, and the lack of proper *CMAS*-deficient *in vitro* or *in vivo* models make it complicated to further explore therapeutic options.

### SLC35A1-CDG

#### Clinical symptoms

The *SLC35A1* gene encodes for the CMP-Sia transporter (CST), responsible for importing CMP-Sia into the Golgi apparatus to support glycosylation (Fig. 1). In SLC35A1-CDG (OMIM#603585), loss-of-function variants impair transporter activity, resulting in hyposialylation of glycoproteins. There are six known patients of SLC35A1-CDG to date, and neurological involvement is a recurring disease phenotype (144, 145, 146, 147, 148, 149). Core manifestations include developmental delay, intellectual disability, seizures, hypotonia, and movement disorders. Microcephaly, ataxia, and involuntary movements have also been noted (144, 145, 147), reflecting both cortical and extrapyramidal involvement. Neuroimaging in two of the patients revealed no major structural anomalies, though functional abnormalities were evident (144, 145). The first reported case in 2001, which showed a severe hematological phenotype, did not have any reported neurological symptoms, though detailed assessment was limited due to early death (146, 148).

#### Genetic spectrum and molecular and biochemical profile

All six patients have been molecularly characterized (Table 4). The first reported case carried a c.147 T > C variant with two microdeletions in the first allele (c.[147T > C;277del;281del], p.(Val93Cysfs∗17)) and an insertion in intron 6 (c.752-157_752-156insCTCA, p.(=)) in the second allele, both of which were predicted to cause a frameshift and premature stop codon. Subsequent studies excluded the role of c.752-157_752-156insCTCA in inducing alternative splicing; however, the combination of one mutated allele and one alternatively spliced nonfunctional allele was sufficient to cause disease (150). The second patient carried homozygous missense variant c.303 G > C (p.Gln101His), causing about a 50% reduction in CST activity and impaired substrate binding (145). p.Gln101 is critical for coordinating CMP-Sia in the binding pocket and replacement with histidine likely disrupts this interaction (145, 151, 152). A recent study further confirmed that p.Gln101His reduces transport function and highlighted that the shorter CST isoform retains more residual activity, suggesting that isoform context may influence disease severity (153).

**Table 4:** All genetic variants identified and documented in SLC35A1-CDG patients

Nucleotide substitution NM_006416.5	Amino acid substitution NP_006407.1	*SLC35A1* exon	SLC35A1 protein domain	Variant type	Clinical classification (ClinVar)	Reference
c.133 A > G	p.Thr45Ala	2	1st luminal loop	Missense	Likely pathogenic/VUS/likely benign	(149)
c.[147T > C;277del; 281del]	p.(Val93Cysfs∗17)	2 and 3	TMD3	Frameshift	Pathogenic	(146, 148)
c.303 G > C	p.Gln101His	3	TMD3	Missense	Pathogenic	(145)
c.439 T > C	p.(Ser147Pro)	4	TMD5	Missense	Pathogenic	(147)
c.467 C > G	p.(Thr156Arg)	4	TMD5	Missense	Pathogenic/likely pathogenic	(144)
c.586 G > A	p.(Glu196Lys)	6	TMD6	Missense	Pathogenic/likely pathogenic	(144)
c.752–157_752–156insCTCA^a^	p.(=)	Intron 6	3rd cytoplasmic domain	Splicing-altering variant	Benign	(148, 150)

TMD, transmembrane domain.

The location of the variant on the exon (or intron) number and on the protein domain are given. An asterisk (∗) indicates a premature stop codon. The clinical classification of the variants are reported based on the Clinical Variation (ClinVar) database.

a Although this variant was initially reported to alter splicing, subsequent studies demonstrated that it was a silent polymorphism (156).

The third patient reported had compound heterozygous variants c.467 C > G (p.(Thr156Arg)) and c.586 G > A (p.(Glu196Lys)), both reducing transport activity by 9-fold and likely affecting conformational changes. Specifically, p.(Thr156Arg) is thought to disrupt CST dimerization, a requirement for transport activity (144, 154). Patients 4 and 5, two siblings from consanguineous parents, possessed homozygous missense variant c.439 T > C (p.(Ser147Pro)) (147). This variant was predicted to be deleterious and likely destabilizes the transmembrane helix or disrupts the adjacent cytoplasmic loop crucial for CST function. The most recently reported SLC35A1-CDG patient carried a homozygous missense variant c.133 A > G (p.Thr45Ala), affecting a conserved residue adjacent to transmembrane domain 2. Functional studies showed reduced CST activity, likely due to disruption of an interhelical hydrogen bond and altered protein flexibility (149).

Aberrant sialylation was observed in all cases *via* biochemical profiling. The first patient showed normal transferrin levels and glycosylation, but a complete lack of sialyl-Lewis^X^ antigens on polymorphonuclear neutrophils (146). Other patients exhibited hyposialylated transferrin with increased asialo-, mono-, or disialo-glycoforms—consistent with impaired N-glycan maturation (144, 145, 147). Remarkably, the effect was more pronounced for α2,3- than α2,6-sialylation (155). ApoC-III sialylation was similarly affected, with reduced sialylated isoforms (145, 146). In the most recent patient, consistent hyposialylation was observed in both fibroblasts and serum, supporting pathogenicity despite the relatively mild clinical presentation (149). Even in cases where transferrin analysis alone was inconclusive, these findings confirm systemic hyposialylation due to defective CMP-Sia transport.

#### Disease models and therapeutic strategies

There are currently no curative treatments available for SLC35A1-CDG, and clinical management remains symptomatic. One patient received speech and physical therapy, along with antiepileptic treatment that was initially effective; however, seizures and cognitive impairment ultimately persisted (144). Recent molecular insights open potential avenues for targeted therapies. A functional SLC35A1 splice variant has been identified, encoding a shorter CST isoform (150). This may be particularly relevant for patients with the p.Gln101His variant, where CST activity in the shorter isoform remains preserved (153). Interestingly, this variant also disrupted O-mannosyl glycosylation of α-dystroglycan in SLC35A1 KO HAP1 cell lines, a phenotype that could be partially rescued by ribitol supplementation, thereby linking CST function to CDP-ribitol homeostasis (47). A recent study corroborated this, revealing that SLC35A1 and its paralog SLC35A4 have redundant roles, and that SLC35A1 can transport both CMP-Sia and CDP-ribitol (48). This suggests a broader metabolic role and raises the possibility of cofactor or substrate supplementation as a potential therapy for patients with certain variants of SLC35A1-CDG.

Although sialylation has been extensively studied in neurodevelopment, models specifically exploring SLC35A1 function in the brain remain limited. Early studies in the 1990s used CHO mutant cell lines lacking SLC35A1 to show disrupted polySia synthesis, which with current knowledge potentially links CST deficiency to NCAM dysfunction and associated neurological symptoms (157, 158). More recently, a relevant disease model demonstrated that inhibiting the Sia biosynthesis pathway in human iPSC-derived excitatory neurons led to defective neuronal network formation, impaired calcium signaling, and altered expression of synaptic markers, providing direct evidence that hyposialylation can perturb neurodevelopment (79). These findings highlight the need for more CNS-relevant models. In contrast, several studies have investigated hematologic phenotypes associated with SLC35A1 mutations; these are discussed in more detail in the **Platelets** section of this review.

### Concluding remarks: Sia metabolism in the brain

Together, the disorders caused by genetic defects in *GNE*, *NANS*, *CMAS*, and *SLC35A1* provide a window into the complexities of Sia metabolism and its essential role in human brain development and function. With the Sia pathway being the common denominator, these conditions present with a diverse spectrum of neurological and biochemical features. GNE, NANS, CMAS, and SLC35A1 are all ubiquitously involved in biosynthesis of sialylated glycoconjugates and manifest with intellectual disability and speech impairment, yet diverge biochemically. In fact, reduced sialylation of serum transferrin was only observed in SLC35A1-CDG patients, raising the question whether NANS-CDG and CMAS deficiency result in brain-specific hyposialylation of gangliosides or polySia *via* unsolved tissue-specific mechanisms. Another possibility might be that transient hyposialylation in these diseases causes early defects in neurodevelopment, which may be missed by analysis of serum glycoprotein markers postnatally. Metabolic consequences of these four defects also differ, with SLC35A1-CDG resulting in reduced sialylation, while GNE sialuria causes hypersialylation. Since the neurological symptoms are rather generic and could be caused by a wide variety of biological mechanisms, it cannot be excluded that these genetic defects result in overlapping neurological symptoms *via* distinct biochemical mechanisms. Supporting this, GNE-related sialuria is marked by excessive free Sia in the urine, increased intracellular CMP-Sia, and hypersialylation of N- and O-glycans, highlighting multiple nodes of dysregulation along the same biosynthetic route. A consistently observed feature across these disorders is CNS involvement, yet detailed understanding of how disturbed sialylation leads to specific neurological deficits remains incomplete. Remarkably, Neu5Gc is almost completely absent in the vertebrate brain, even in organisms that retained the ability to *de novo* synthesize Neu5Gc (159). This suggests a possible toxicity of Neu5Gc in the brain and emphasizes the importance of physiological impacts of different Sia types. Moreover, a possible physiological role of KDN in the brain is largely unexplored. It is also unknown whether distinct Sia linkages or modifications differentially impact neural circuits or how regional and cell type-specific sialylation patterns are established and maintained. Further research, particularly in human-relevant neuronal models, is urgently needed to elucidate the nuanced regulation and functional significance of Sia metabolism in brain development and disease.

## Disorders of Sia metabolism affecting the muscle

Sias are present in different types of glycoconjugates and have several roles in muscle development and function. The importance of Sias in skeletal muscle function is evident from genetic disorders that affect Sia metabolism genes such as *GNE* and *NPL*, both of which lead to myopathies (72, 160). NPL deficiency can also lead to cardiomyopathy (72), whereas cardiac involvement is not clearly associated to GNE myopathy (160). Embryonic lethality of *Gne* KO mice shows the essential role of sialylation in development (161). Impaired skeletal muscle development was observed in studies with embryonic stem cells from *Gne* KO mice and g*ne* KD zebrafish, further emphasizing the role of Sia in skeletal muscle development and function (162, 163).

Sialylation is a common feature of muscle proteins, such as α-dystroglycan, neprilysin, and voltage-gated ion channels (164, 165, 166). In humans, Neu5Ac has been detected during fetal skeletal muscle development (167). PolySia is present in the early stages of muscle development and decreases over time (167). As myoblasts fuse into multinucleated cells which eventually give rise to muscle fibers (168), the timing of polySia expression is important for proper myoblast differentiation. Expression during the early differentiation stage inhibits myoblast fusion (169), whereas later stage expression of polySia is thought to terminate further cell fusion and promote the separation of myotubes (167, 169). These effects are likely due to the strong negative charge of polySia which repels cell membranes (170) and may be further amplified when polySia is bound to fibroblast growth factor 2, a known inhibitor of myoblast differentiation (171, 172). As polySia expression declines, monomeric α2,3- and α2,6-linked Sias begin to emerge, followed by the appearance of acetylated Sia during fetal development (167). These acetylated Sias are more resistant to cleavage by sialidases (173), which play important roles in skeletal muscle as extensively reviewed by Fanzani *et al*. (174).

Beyond protein glycosylation, gangliosides also contribute to muscle biology. As previously mentioned, gangliosides are most abundant and well-studied in the brain (6, 78); however, emerging evidence suggests they may also contribute to skeletal muscle development and physiology (175, 176, 177, 178, 179, 180). For example, during murine C2C12 myoblast differentiation, a transition occurs on GM3 gangliosides from Neu5Ac to Neu5Gc, influencing cell adhesiveness and myogenesis (180, 181). Since humans lack endogenous Neu5Gc and therefore murine mechanisms cannot be directly translated, a *Cmah*^−/−^ mouse model has been generated to resemble human-like sialylation (182). One study observed that the lack of Neu5Gc in these *Cmah*^*−/−*^ mice had a positive effect on exercise endurance (183). In addition, these *Cmah*^*−/−*^ mice have been used to model human muscular dystrophies, among which Duchenne muscular dystrophy and limb girdle muscular dystrophy 2D. Interestingly, the disease severity of both dystrophies was increased when modeled in a *Cmah*^*−/−*^ background compared to using a *Cmah*^*+/+*^ background (184, 185). This effect was likely due to increased anti-Neu5Gc immunity (186). These examples illustrate how Sia can differentially influence multiple muscular processes, once again highlighting complex cell- and tissue-specific effects.

Sialylation patterns continue to evolve in adult and aging skeletal muscle. Different linkage forms of Sia have been detected in adult skeletal muscle tissue (187, 188, 189). In muscle, Sias were predominantly bound to glycans, with free Sias representing only a small fraction (190). With age, monomeric and acetylated Sias disappeared, while an increase in polySia was observed (188), possibly related to denervation and re-innervation processes in aging muscle (191, 192). Moreover, sialylation has been shown to be reduced in damaged muscles, suggesting a role in muscle repair and regeneration (193). Altogether, Sia plays several roles in muscle development and function, and this is disrupted by defects in Sia metabolism. The following sections will explore the muscle-related disorders, GNE and NPL myopathy, in Sia metabolism in more detail.

### GNE myopathy

#### Clinical symptoms

The first signs of GNE myopathy (OMIM#605820) usually appear in early adulthood, often presenting as foot drop, caused by progressive distal muscle weakness (160). As the disease progresses, muscle weakness gradually extends to the proximal limbs, and most patients ultimately require a wheelchair for mobility. Interestingly, the quadriceps remain relatively spared. Cardiac and respiratory muscles are generally not involved but may occur at an advanced disease stage (194). Additional clinical features may include sleep apnea and thrombocytopenia (195), the latter of which is discussed in more detail in the **Platelets** section. Furthermore, the presence of rimmed vacuoles are characteristic for muscle biopsies of GNE myopathy patients.

#### Genetic spectrum

GNE myopathy is an autosomal recessive disease. Currently, a total of 376 genetic variants have been associated with GNE myopathy (196). Seven prevalent founder variants have been identified in specific populations, including p.Asp207Val and p.Val603Leu in Japan, p.Asp409Tyr and p.Ala662Val in the United Kingdom, p.Ile618Thr in the Roma population in Bulgaria, p.Val727Met in India, and p.Met743Thr in the Middle East (160). Notably, no patients have been diagnosed with biallelic null variants, suggesting that a minimum level of residual GNE activity is necessary for development.

Many hospital-based and population studies have demonstrated compelling genotype–phenotype correlations. For example, the p.Asp207Val variant is commonly associated with later disease onset, milder symptoms, and slower progression, while other variants, such as p.Val603Leu, tend to result in earlier onset and a more severe phenotype (197, 198). Additionally, genetic variants confined to the kinase domain, particularly in a homozygous state, were linked to a more aggressive phenotype than compound heterozygous variants affecting both the epimerase and kinase domains (199, 200). Nevertheless, considerable variability remains in clinical presentation, even among carriers of identical mutations, underscoring the broad influence of aberrant Sia metabolism in muscles.

#### Molecular and biochemical profile

*GNE* mRNA expression is relatively low in skeletal muscles compared to other tissues (201, 202). Protein expression has been detected in cultured fetal human muscle cells but was barely present in adult human skeletal muscle tissue (203). Correspondingly, GNE expression in mice was low in skeletal muscle and further declined during maturation (204), while GNE was upregulated during muscle damage and regeneration (205). In patients, GNE expression is affected minimally, and decreased enzyme activity is considered to play a more significant role underlying the development of myopathy (206, 207). Interestingly, some variants may still retain a relatively high residual enzymatic activity in one or both domains (208).

Whether reduced enzyme activity also results in hyposialylation is still inconclusive and may differ among tissues and the type of glycosylation. While sialylation of serum transferrin and ApoC-III is not abnormal (209, 210), hyposialylation has been detected in muscle tissue sections stained by lectins, and hyposialylated muscle glycoproteins have been detected, *i*.*e*.*,* α-dystroglycan and NCAM (46, 211, 212). Furthermore, decreased free Sia levels were detected in serum and muscle tissue of GNE myopathy patients (190). However, other reports do not clearly support the finding of overall hyposialylation, which was, for example, the case in patient-derived muscle cells or lymphoblastoid cells (187, 213). In addition, Saito *et al*. only detected muscle protein hyposialylation in one patient with genetic variants in the epimerase domain but not in three other patients with variants in both domains or only in the kinase domain (214). In general, the sample size in these studies was low and the discrepancies between studies could additionally be explained by varying methodologies, differences across genetic variants, or interpersonal variation in possible compensatory mechanisms.

Currently, few studies have investigated the direct effect of impaired GNE activity in patient material on the metabolic products of GNE, which are ManNAc and ManNAc 6-phosphate. ManNAc has been suggested to be alternatively phosphorylated by NAGK, which could act as a compensation mechanism for the lack of GNE kinase activity in unaffected tissues (42). Possible tissue specificity is further highlighted by the finding that ManNAc restored sialylation defects in a human embryonal kidney (HEK) GNE KO cell model, but failed to do so in a murine myoblast Gne KO cell model, even though *NAGK* was expressed in both cell lines (215, 216). Therefore, further research is required to provide definitive evidence on a potential compensation mechanism by NAGK.

The disease mechanism causing GNE myopathy has not been resolved yet and is further complicated by evidence of additional functions of GNE in the cell unrelated to Sia biosynthesis. This has recently been reviewed by Pereira *et al*. and includes functions related to endoplasmic reticulum stress, oxidative stress, and cytoskeletal organization (217). Taken together, more research is needed to fully comprehend the disease mechanism.

#### Disease models

Since a *Gne* KO is embryonically lethal in mice, several attempts have been made to introduce pathogenic *GNE* variants (161). A transgenic mouse model was developed that expresses the D176V protein variant (corresponding to p.(Asp207Val) in transcript NM_001128227.3) in a *Gne* null background (218). Although this initial transgenic mouse model showed hyposialylation and a muscle phenotype, the model exhibited an inconsistent phenotype when used by other laboratories, raising concerns about its reproducibility and reliability (219, 220). An additional key limitation of transgenic mouse models is the overexpression of genes, which may not accurately reflect the natural expression levels found in humans. A knock-in mouse model was generated in which the M712T variant [corresponding to p.(Met743Thr) in transcript NM_001128227.3] was introduced (221, 222, 223). These mice showed phenotypes ranging from severe renal dysfunction, mild symptoms, or asymptomatic. Muscle function however was not impaired; thus, the model was not able to recapitulate GNE myopathy. Similarly, knock-in mice carrying the GNE V572L variant [corresponding to p.(Val603Leu) in transcript NM_001128227.3] presented with renal abnormalities and had a short lifespan (224). Moreover, a Gne^FLAG^ mouse model has been developed, which enables detection of Gne distribution over time (204). This model was subsequently used to develop an inducible muscle *Gne* KO model and a double tissue muscle and liver *Gne* KO model, but both models did not exhibit muscle symptoms (225); therefore, a whole body conditional *Gne* KO model is being developed. Overall, a GNE myopathy mouse model that represents muscle symptoms has yet to be developed. Furthermore, zebrafish have been used to generate a *gne*-deficient model using morpholinos to KD *gne* or CRISPR/Cas9 to KO *gne* (163, 226). An abnormal skeletal muscle myofiber organization was observed in both models, which ultimately resulted in early death in the *gne* KO zebrafish. KD of *gne* resulted in a high mortality rate and a variety of disease severity, while transgenic zebrafish expressing human *GNE* cDNA carrying the M743T variant have completely normal development (226). The high early mortality makes these zebrafish models less suitable to study disease progression. *In vitro* approaches have focused on developing a GNE-deficient skeletal muscle cell model derived from iPSCs (227, 228). Taken together, a reliable disease model that is able to recapitulate GNE myopathy remains to be established and is crucial for further functional and preclinical studies to aid the development of effective treatments.

#### Therapeutic strategies

Since a global treatment is not yet available for GNE myopathy, treatment of patients primarily focuses on managing symptoms and maintaining quality of life (160). Multiple clinical trials have been conducted for GNE myopathy, mainly focusing on restoring sialylation, and several recent reviews have summarized these trials (217, 229, 230). The first clinical pilot study was a treatment with intravenous immune globulin (NCT00195637), a highly sialylated glycoprotein (210). Although mild improvements in muscle strength were observed, increased muscle sialylation could not be detected. The first phase 1 clinical trial tested oral Sia supplementation (NCT01236898), but Sia was likely excreted too rapidly *via* the urine to be effective (231). To address this, an extended-release formulation of Sia (Ace-ER) was developed, which increased free serum Sia levels without causing significant side effects in phase 1 trials (NCT01359319) (231, 232). Subsequently, the phase 2 trial showed that muscle strength was stabilized (NCT01517880) (233); however, the international phase 3 trial (NCT02377921) did not show improved muscle strength in patients compared to the placebo, thereby failing to confirm Ace-ER efficacy (234). In parallel, Japanese clinical trials, including a phase 2/3, phase 3 (NCT04671472), and phase 3 extension study, reported more beneficial outcomes regarding maintenance of muscle strength, ultimately leading to the approval of Ace-ER in Japan as the first treatment for GNE myopathy (235, 236, 237). Discrepancies in study populations, including sample size, baseline disease progression, and genetic background, may account for the different outcomes between the two phase 3 clinical trials.

Alternative methods to provide Sia have been explored. One approach is through 6′-sialyllactose, a natural human milk oligosaccharide which has a prolonged circulation time compared to free Sia. 6′-sialyllactose supplementation was first tested in a transgenic GNE myopathy mouse model which resulted in reduced disease progression (238). A follow-up randomized pilot trial in patients demonstrated that 6′-sialyllactose was safe and well tolerated (239). A complementary placebo-controlled study showed that 6′-sialyllactose supplementation significantly slowed down fat replacement in the posterior thigh, but improvements in muscle strength were unfortunately limited (240). Another avenue under investigation in clinical trials to restore sialylation is the administration of the Neu5Ac biosynthesis precursor ManNAc. Since ManNAc is a neutral molecule, it is expected to enter cells more efficiently than Sia. Phase 1 (NCT01634750) and phase 2 trials (NCT02346461) showed that ManNAc supplementation was safe and well tolerated, resulting in only some gastrointestinal complaints (241, 242). Furthermore, increased ManNAc and Sia levels in the plasma were observed (241, 242). A phase 2 multicenter study (NCT04231266) is currently in progress to further evaluate efficacy.

Finally, gene therapies have become a promising treatment strategy for several neuromuscular disorders, with approved drugs already available for Duchenne muscular dystrophy and spinal muscular atrophy (243, 244). Application of gene therapies to GNE myopathy are being explored, mainly in preclinical models and one study included a single GNE patient (219, 220, 245, 246, 247). The lack of an effective therapy for GNE myopathy could be attributed to the absence of a reliable animal model required for preclinical studies and the complexity of the disease mechanisms that remains poorly understood, including whether reduced sialylation is really the cause behind myopathy. Additionally, finding a proper biomarker would be a valuable addition to monitor treatment efficacy next to clinical evaluations. This may also help in better understanding and optimization of the pharmacodynamics of ManNAc, which is showing some positive effects in patient trials.

### NPL myopathy

#### Clinical symptoms and genetic spectrum

NPL is the key enzyme involved in the first step of Sia catabolism (Fig. 1). While its function has been extensively studied in bacteria, its role in humans received little attention until its association with a genetic disease resulting in NPL myopathy was first described in 2018 (72). To date, only one sibling pair with variants in the *NPL* gene has been described in literature, of which only one sibling participated in the full clinical examination (72). Compound heterozygous missense variants were identified: c.187 C > T (p.Arg63Cys) and c.133 A > G (p.Asn45Asp). Given the recent discovery of the role of NPL in disease, additional NPL patients are likely still undiagnosed. Clinical symptoms observed in the siblings include high urinary Neu5Ac, muscle weakness, and exercise intolerance. Progressive dilated cardiomyopathy was also observed in one of the siblings, with cardiac complications already identified prenatally and muscular symptoms later documented at 16 years of age.

#### Molecular and biochemical profile

In humans, *NPL* gene expression is most abundant in the spleen, with comparatively lower expression levels in the heart and skeletal muscle (72). In mice, NPL enzyme activity was highest in the colon, kidney, and spleen, while the heart and skeletal muscle exhibited a relatively lower activity (248). During zebrafish embryonic development, *npl* already showed expression in the early stages, including in somites and embryonic muscles (72). Interestingly, a recent study revealed that NPL levels in skeletal muscle positively correlated with fatigue resistance, suggesting a functional role for NPL in maintaining muscle endurance and health (249). These findings imply that even at low expression levels, NPL activity in muscle tissue may contribute to metabolic pathways essential for muscle performance, and its dysregulation could impact muscle function.

NPL orthologs from various organisms display broad substrate specificity and can cleave different types of Sias (250, 251), with a preference for the open form of its substrate over the cyclic form (252). In humans, NPL recognizes both Neu5Ac and Neu5Gc as substrates, with higher affinity for Neu5Ac (71, 72). The two patient missense variants identified have been studied *in vitro* (72, 253). Protein expression and stability of the p.Arg63Cys variant was largely reduced in one study (72) and could not be detected at all in a second study, likely due to protein insolubility (253). The p.Asn45Asp variant also showed a reduction in protein expression, protein stability, and enzymatic activity (72, 253). At the biochemical level, sialylation of transferrin and apoC-III appeared normal in the NPL myopathy patient (72). Furthermore, Neu5Ac accumulated in patient red blood cells, while levels of the NPL products *N*-acetylhexosamine and ManNAc 6-phosphate were reduced, consistent with a lack of Neu5Ac catabolism due to absent NPL activity. ManNAc levels were also low in urine and plasma samples. Interestingly, these biochemical defects were not detected in patient fibroblasts, further highlighting tissue-specific mechanisms.

#### Disease models and therapeutic strategies

Two animal models have been developed to study NPL deficiency (72, 248). The first model, in zebrafish, demonstrated that KD of *npl* affected both muscle and heart development and increased reactive oxygen species levels (72). Supplementation with ManNAc successfully rescued the disease phenotype. Injection with human *NPL* mRNA was also able to rescue the phenotype. Other tested supplements, including GlcNAc and Neu5Ac, were less efficient than ManNAc. In the second NPL-deficient model, mice were generated by expressing either the patient variant Arg63Cys or by introducing a 116-bp deletion in exon 4, resulting in a full *Npl* KO (248). Both models showed clear effects on muscle structure and function, further emphasizing the essential function of NPL in muscle. In contrast to the zebrafish model, no involvement of the heart was observed. Interestingly, an increased ratio of Neu5Gc in glycans was observed in the muscle tissue of NPL-deficient mice. Supplementation with ManNAc was able to rescue several defects but did not reverse the accumulation of free Sias (248). Although the mechanism behind rescue with ManNAc is not fully understood, it is an interesting treatment possibility to further explore for NPL myopathy patients.

### Concluding remarks: Sia metabolism and muscle

While the molecular mechanisms are still not well understood, these findings highlight the crucial role of Sia metabolism in skeletal muscle development, maintenance, and disease. Despite their contrasting roles in Sia metabolism, variants in both the biosynthetic GNE enzyme and the catabolic NPL enzyme affect the muscle, suggesting potential overlap in the underlying biochemical mechanisms driving the diseases. Defects in NPL clearly lead to accumulation of Neu5Ac in urine and red blood cells (72), while Sia levels are low to normal in serum of GNE myopathy and decreased in muscle. Thus far, muscle biopsies from NPL myopathy patients have been inaccessible for further investigations. Metabolic overlap is possibly present in the low levels of ManNAc and ManNAc 6-phosphate, as detected in materials of NPL myopathy. These markers have not been detected before for GNE myopathy but are expected to also be low, since they are the direct products of GNE enzymatic activity. Although both defects result in a muscle disease, the exact clinical symptoms differ. A striking feature is the late onset of muscle symptoms in GNE myopathy, despite its congenital genetic basis, raising the question of whether Sia deficiency alone is sufficient to trigger disease. GNE myopathy also typically spares cardiac muscle until more advanced stages, whereas NPL deficiency manifests earlier in patients and includes the heart. These differences occur despite relatively low expression of both genes in skeletal muscle, suggesting that even minimal enzymatic disruption can have significant downstream effects or that Sia imbalance indirectly affects muscle integrity through broader metabolic signaling pathways. Similarly, the low expression of NPL in muscle contrasts with the severity of the phenotype observed upon its loss, suggesting that even modest perturbations in Sia turnover has significant consequences. Interestingly, ManNAc has been explored as a treatment for both diseases and is expected to restore hyposialylation in GNE myopathy. However, the mechanism behind successful treatment of NPL-deficient mice with ManNAc is not clear. Perhaps ManNAc serves additional functions in skeletal muscle beyond Sia metabolism. While the link between sialylation and muscle pathology is well established, the precise mechanism by which its disruption leads to disease remains only partially understood.

## Disorders of Sia metabolism affecting platelet function and clearance

Platelets are small, enucleated cell fragments that circulate in the blood to prevent and stop bleeding, and defects may occur when Sia metabolism is deficient. During thrombopoiesis, megakaryocytes first generate proplatelets, which eventually mature into platelets (254). Sia plays a crucial role in platelet biology. Platelets have been described as a potential source of sialyltransferases and their donor substrate CMP-Sia (255, 256, 257). Sialyltransferases are responsible for sialylation, which is required for proper megakaryocyte differentiation, proplatelet formation, and regulation of platelet lifespan (258, 259, 260). As an example, platelet glycoprotein Ibα (GPIbα) is highly sialylated and becomes desialylated over time by NEUs (261). The resulting exposure of the underlying galactose and GlcNAc residues make such glycoproteins recognized as substrate by the Ashwell-Morell receptor on hepatocytes or by receptors on liver macrophages called Kupffer cells (261, 262). This coordinated process ensures the removal of aged, desialylated platelets from circulation (263, 264).

Given the critical role of Sia in platelet function and lifespan, it is not surprising that defects in Sia biosynthesis lead to platelet disorders. Patients with genetic defects in *NANS*, *GNE*, and *SLC35A1* may exhibit a low platelet count, also known as thrombocytopenia, and macrothrombocytopenia when platelets are also enlarged. As a result, patients have an increased tendency to bleed or bruise and an increased risk of hemorrhage after surgery or injury. In the section below, each disorder is discussed in more detail.

### GNE thrombocytopenia

#### Clinical symptoms

Thrombocytopenia was first reported as a symptom in two GNE myopathy patients in 2014 (265), and later studies showed that thrombocytopenia may also occur independently from myopathy (266, 267, 268). Severe thrombocytopenia was observed in most patients, which is characterized by a platelet count of <50 x 10^9^/L. Four patients had a count between 50 and 99 x 10^9^/L, which is classified as moderate thrombocytopenia, and one patient with a platelet count of 103 x 10^9^/L had mild thrombocytopenia (269, 270, 271). Specifically, the subtype of macrothrombocytopenia was consistently observed in patients (266, 272, 273), and first symptoms usually manifest early in childhood (274, 275, 276). Some studies report immature megakaryocytes (272, 276, 277), and others report an increased immature platelet fraction, suggesting a high platelet turnover (266, 278). In general, other blood cell types are not affected, though leukopenia and neutropenia have been observed in some patients (269, 278, 279). Interestingly, one case additionally presented with neurological symptoms, which is typically not observed in other patients (275).

#### Genetic spectrum

In literature, a total of 42 different genetic variants have been identified among 53 patients with GNE thrombocytopenia, either with or without myopathy. Some variants, *i*.*e*.*,* p.Cys44Ser and p.Val603Leu, have previously been associated with GNE myopathy patients (208), but multiple novel GNE variants have been described as well, which appear to be exclusively associated with isolated thrombocytopenia. Previously, Bottega *et al*. identified the kinase domain as a mutational hotspot, particularly the residues involved in binding of ADP and ManNAc (272). Therefore, it was hypothesized that megakaryocytes and platelets are especially vulnerable to impaired kinase activity. Table 5 summarizes all genetic variants detected in GNE thrombopenia patients reported in literature so far. Fifteen of the described genetic variants were located in the epimerase domain, of which seven impact the kinase domain as well due to a nonsense variant or frameshift resulting in protein truncation. In addition, a deep-intronic variant (c.862 + 870C > T) was detected, which introduced a pseudo-exon between exon 5 and 6, thereby predicted to disrupt the reading frame and yield a truncated protein (280). This truncated protein was predicted to affect binding at the epimerase domain while the kinase domain was completely lost. From the remaining variants, 23 variants were located in the kinase domain, and two variants were in a region of unknown function located between the epimerase and kinase domains. Both of these variants were predicted to result in a truncated protein in which the kinase domain was lost. In the case of the intronic variant (c.1163+5G > T), the kinase domain was predicted to be lost due to exon 6 skipping (281).

**Table 5:** All genetic variants identified and documented in GNE thrombocytopenia patients

Nucleotide substitution NM_001128227.3	Amino acid substitution NP_001121699.1	*GNE* exon	GNE protein domain	Variant type	Clinical classification (ClinVar)	Reference
c.115 C > T^a^	p.(Arg39∗)	2	Epimerase	Nonsense	Pathogenic/likely pathogenic	(282)
c.125 G > A	p.(Arg42Gln)	2	Epimerase	Missense	Pathogenic/likely pathogenic/VUS	(270)
c.131 G > C^a^	p.(Cys44Ser)	2	Epimerase	Missense	Pathogenic	(195)
c.268 C > T^a^	p.(Arg90∗)	3	Epimerase	Nonsense	Pathogenic	(280)
c.304 A > T^a^	p.(Arg102Trp)	3	Epimerase	Missense	Likely pathogenic/VUS	(269)
c.395 G > A^a^	p.(Arg132His)	3	Epimerase	Missense	Pathogenic/likely pathogenic/VUS	(195)
c.416_426del	p.(Ile139Argfs∗4)	3	Epimerase	Frameshift	Not classified	(278)
c.476dup^a^	p.(Arg160Profs∗6)	3	Epimerase	Frameshift	Not classified	(282)
c.478 C > T^a^	p.(Arg160∗)	3	Epimerase	Nonsense	Pathogenic/likely pathogenic	(280, 283, 284)
c.562 C > T	p.(His188Tyr)	3	Epimerase	Missense	VUS	(266)
c.649 T > C^a^	p.(Tyr217His)	3	Epimerase	Missense	Pathogenic	(265)
c.705 G > A^a^	p.(Trp235∗)	3	Epimerase	Nonsense	Not classified	(285)
c.787del^a^	p.(Met263Cysfs∗4)	4	Epimerase	Frameshift	Pathogenic/likely pathogenic	(286)
c.812 T > A	p.(Leu271His)	4	Epimerase	Missense	Not classified	(280)
c.862 + 870C > T^a^	p.(Gly288Alafs∗4)	Intron 4	Epimerase	Splice-altering variant	Not classified	(280)
c.1163+5G > T	p.(Gly359Valfs∗15)	Intron 6	UF	Splice-altering variant	Not classified	(281)
c.1277del	p.(Asn426IleIfs∗13)	7	UF	Frameshift	Not classified	(281)
c.1330 G > T	p.(Asp444Tyr)	7	Kinase	Missense	Likely pathogenic	(276)
c.1339 G > A	p.(Gly447Arg)	7	Kinase	Missense	VUS	(275)
c.1340 G > A	p.(Gly447Glu)	7	Kinase	Missense	Not classified	(268)
c.1343 C > T	p.(Thr448Met)	7	Kinase	Missense	VUS	(277, 286)
c.1351 C > T^a^	p.(Arg451∗)	7	Kinase	Nonsense	Pathogenic	(195, 276, 282)
c.1352 G > A	p.(Arg451Gln)	7	Kinase	Missense	VUS	(273, 277, 278)
c.1516_1517 delinsTT	p.(Gly506Phe)	9	Kinase	Missense	Not classified	(266)
c.1546_1547 delinsAG	p.(Val516Arg)	9	Kinase	Missense	Not classified	(272)
c.1550 T > C	p.(Leu517Pro)	9	Kinase	Missense	Likely pathogenic	(266)
c.1636_1637del^a^	p.(Asp546Glnfs∗2)	9	Kinase	Frameshift	Pathogenic/likely pathogenic	(265)
c.1649 A > G^a^	p.(Asn550Ser)	9	Kinase	Missense	Pathogenic/likely pathogenic	(266)
c.1664 C > T^a^	p.(Ala555Val)	9	Kinase	Missense	Pathogenic/likely pathogenic	(195)
c.1724 C > G	p.(Thr575Arg)	9	Kinase	Missense	Not classified	(272)
c.1727 G > C	p.(Gly576Ala)	10	Kinase	Missense	Not classified	(287)
c.1732 G > A	p.(Gly578Ser)	10	Kinase	Missense	VUS	(267, 285, 288)
c.1768 G > A^a^^,^^b^	p.(Gly590Arg)	10	Kinase	Missense	Pathogenic/likely pathogenic/VUS	(275, 289)
c.1781 G > A	p.(Cys594Tyr)	10	Kinase	Missense	Not classified	(290)
c.1807 G > C^a^	p.(Val603Leu)	10	Kinase	Missense	Pathogenic	(195, 274, 282, 284)
c.1864 G > A^a^	p.(Ala622Thr)	10	Kinase	Missense	Pathogenic/likely pathogenic/VUS	(195)
c.1900C > T^a^	p.(Leu634Phe)	10	Kinase	Missense	Pathogenic/likely pathogenic/VUS	(270)
c.1937C > G^a^	p.(Ser646∗)	11	Kinase	Nonsense	Pathogenic	(269)
c.2096C > T	p.(Ser699Phe)	12	Kinase	Missense	Likely pathogenic/VUS	(279)
c.2204 C > G	p.(Pro735Arg)	12	Kinase	Missense	Not classified	(272, 290)
c.2215 G > A^a^	p.(Gly739Ser)	12	Kinase	Missense	Likely pathogenic	(274)
c.∗1012CA (13)^c^	p.(=)	12	-	3′UTR variant	Not classified	(283)

The nucleotide variants are reported based on the transcript reference sequence NM_001128227.3, and protein variants are reported based on the protein reference sequence NP_001121699.1, following previous recommendations (291). The location of the variant on the exon (or intron) number and on the protein domain are given. The clinical classification of the variants are reported based on the Clinical Variation (ClinVar) database. 3′UTR: 3′ untranslated region; UF, unknown function; VUS, variant of uncertain significance.

a This genetic variant has also been observed in patients with GNE myopathy.

b The authors did not consider the c.1768 G > A variant in GNE as the causative variant of thrombocytopenia, as an alternative variant in the PRKACG gene was considered a stronger disease-causing candidate.

c c.∗1012CA (13) is in alignment with the sequence of the human reference genome (GRCh38), thus interpretation of the pathogenicity should be taken with caution.

#### Molecular and biochemical profile

GNE protein expression was shown to be reduced for the variants p.Val516Arg, p.Thr575Arg, and p.Pro735Arg (272, 290). However, it is likely that decreased enzymatic activity plays a more prominent role in disease. Several variants were predicted to disrupt the binding site with ADP or ManNAc, thereby impacting GNE kinase activity. The residues p.Asp444, p.Gly447, p.Thr448, p.Arg451, and p.Thr575 are all located in the ADP-binding pocket, while p.Gly506 and p.Gly578 are near the binding site of the substrate ManNAc (272, 277). In addition, the variants p.Val516Arg, p.Leu517Pro, p.Cys594Tyr, and p.Pro735Arg were predicted to destabilize folding of the kinase domain (272, 290).

The effect of impaired GNE kinase activity on glycan sialylation has been extensively studied (272). One study used a metabolic labeling strategy to demonstrate reduced sialylation for the variants p.Cys594Tyr and p.Pro735Arg, likely due to the loss of kinase function (290). One patient showed slightly reduced free Sia levels in serum (283). Interestingly, sialylation of serum transferrin and apoC-III was detected as normal in two patients (273), while a decrease in tetrasialo-transferrin was detected in two patients from another study (272). In platelets, both α2,3- and α2,6-linked Sias appeared to be reduced based on lectin staining (266, 267, 278, 290). A decrease in cell surface sialylation was also seen in white blood cells (278) but not for red blood cells (286). In two studies, a significant reduction of α2,3-linked Sias was observed concomitantly with an increase of galactose residues on the platelet cell surface, suggesting that the α2,3-linkage is more affected (273, 277). Remarkably, one study showed a clear reduction of α2,6-linked Sia, while no difference could be observed in galactose expression on platelets (286). The discrepancies between these studies could be attributed to several factors, including the small sample size, the use of different methodologies (*i*.*e*.*,* lectin combinations), different genetic backgrounds, and variation in disease severity. Although the impact on sialylation has been widely studied, limited studies have determined the direct impact of deficient GNE kinase activity on Sia metabolites in patient material. In addition to the study of serum Sia, only one other study measured free Sia, of which its excretion in urine appeared normal (273).

Subsequently, reduced platelet sialylation increases the susceptibility of platelets to be cleared in the liver. This was supported by the finding that platelets from a GNE thrombopenia patient showed increased binding to THP-1 macrophages and HepG2 hepatocytes, both cell types involved in platelet clearance (273). Furthermore, a short platelet half-life and hepatic sequestration were observed (273). Normal thrombopoietin levels in patients indicated that platelet production was not affected (273, 286). Taken together, the mechanism in GNE thrombopenia could be explained by reduced GNE activity, possibly with a major effect on the kinase domain, resulting in less sialylation of platelets and thereby increased removal from the circulation, rather than impaired platelet production.

#### Disease models and therapeutic strategies

To study genetic variants causing GNE thrombocytopenia, a knock-in mouse model was generated carrying the patient variant p.(Pro735Arg) (290). During embryonic development, these mice had a decreased number of embryonic megakaryocytes and platelets and experienced severe hemorrhages followed by death. It was suggested that a lack of core 1 O-glycan sialylation could explain the disease phenotype in these mice.

Several treatment strategies commonly used to treat immune thrombocytopenia have also been applied to GNE thrombocytopenia patients (292). Corticosteroids suppress immune-mediated platelet destruction, while stimulating platelet production and NEU inhibitors act by preventing platelet desialylation but both were not effective in the case of GNE thrombocytopenia (277, 278, 279). Thrombopoietin receptor agonists have been used to promote platelet production in megakaryocytes, resulting in little to no increase of platelet counts (272, 273, 277, 278, 279, 286). Platelet transfusions are currently the most reliable treatment to temporarily increase platelet counts (275, 278, 286). Furthermore, two patients received a hematopoietic stem cell transplantation, which successfully normalized platelet counts in one patient but was fatal in a second patient due to complications (277, 279). Since thrombocytopenia is usually not life-threatening, medication may only be necessary in high-risk scenarios, such as surgeries and severe bleeding events (272). Currently available treatment strategies have failed to resolve thrombocytopenia in the long-term because they do not address the genetic defect underlying platelet hyposialylation. GNE thrombocytopenia patients may benefit from therapeutic strategies currently under development for GNE myopathy. In fact, a clinical trial for GNE thrombocytopenia has been initiated with Ace-ER (NCT02845609); however, efficacy has yet to reported.

### NANS-CDG

Thrombocytopenia was not reported in the first description of NANS-CDG; however, in later reports, variable presence and severity of thrombocytopenia was observed in eight patients (93, 96, 97, 99). This included four cases with severe thrombocytopenia, three with mild thrombocytopenia, and one case that was resolved at a later age. Interestingly, a high level of excreted ManNAc was correlated with a low platelet count (96). In the Sia trial in NANS-CDG patients, Neu5Ac supplementation did not normalize platelet counts (106). Furthermore, giant platelets were mentioned in two patients (99), while bone marrow morphology was either normal or not monitored in the remaining patients (93, 96, 97, 99). Moreover, one patient showed an elevated immature platelet fraction and higher platelet-associated IgG levels (99). The consequences of NANS defects on biochemical parameters have been discussed in the **Brain** section. Thus far, no platelet-specific studies on protein sialylation or Sia metabolites have been reported for NANS-CDG patients in literature.

### SLC35A1-CDG

#### Clinical symptoms

In addition to the extensive neurological symptoms reported in SLC35A1-CDG patients discussed in the **Brain** section of this review, four out of six patients also presented with macrothrombocytopenia, and the most recently diagnosed patient displayed thrombocytopenia in early childhood (144, 145, 146, 147, 148, 149). The reduced platelet count, ranging from 15 x 10^9^/L to 99 x 10^9^/L, contributed to hemorrhagic symptoms such as easy bruising and prolonged bleeding, often leading to complications after surgery (145, 146, 147). Bone marrow smears revealed abnormal megakaryocyte morphology and an accumulation of immature megakaryocytes, pointing to disrupted thrombopoiesis (146, 147, 148). Consistent with impaired cellular maturation, patients also exhibited an increased immature platelet fraction (147).

#### Disease models and therapeutic strategies

As with all the diseases discussed in this review, current interventions have been focused on managing symptoms. The first reported patient received corticosteroids and platelet transfusions to increase platelet count, but the response was transient. The patient received a bone marrow transplant at 34 months of age but died shortly thereafter due to complications (146). The second patient passed away at 22 years of age following surgical complications, with no specific long-term therapeutic strategies reported (145).

Developing relevant disease models continues to be an important step in identifying and improving current therapies. In contrast to the lack of CNS-relevant models for SLC35A1 deficiencies, there have been more studies aimed at exploring the hematological implications. In common cell lines, such as CHO, HEK, and HeLa cells, complete depletion of SLC35A1 resulted in severe general sialylation defects (148, 153, 158, 293), and several mouse models have been explored which recapitulated the sialylation defects observed in humans. A murine *Slc35a1* KO in megakaryocytes and platelets phenocopied many features of human disease, including macrothrombocytopenia, increased immature platelets, and impaired thrombopoiesis (259). While platelets retained some sialylation, likely due to uptake of exogenous sialylated proteins, the incorporation of Neu5Gc was reduced in platelets and megakaryocytes of *Slc35a1*-deficient mice. Interestingly, surface expression of GPIbα was significantly reduced on platelets but unaffected in megakaryocytes, suggesting differential regulation during maturation. Another *Slc35a1* KO mouse model generated in endothelial/hematopoietic cells also exhibited macrothrombocytopenia; however, the liver was severely affected in these mice due to aberrant vascular endothelial growth factor receptor 2 signaling, resulting in premature death (294). Liver symptoms have not been reported in SLC35A1-CDG patients, making this a less suitable model. *Ex vivo* studies using platelets from an SLC35A1-CDG patient further demonstrated that SLC35A1 was required for maintaining platelet life span but was not involved in proplatelet formation in megakaryocytes (147), indicating that enlarged platelets may be a compensatory mechanism to thrombocytopenia.

Interestingly, ST3Gal-IV-deficient mice exhibited spontaneous thrombocytopenia due to reduced α2,3-linked sialylation on platelet glycoproteins (295) and exposure of galactose residues that triggered accelerated platelet clearance (258). Though these mechanisms may differ from SLC35A1, recent reports show that SLC35A1 deficiency and ST3Gal-IV deficiency affect similar glycan structures, indicative of shared pathways and overlapping phenotypes (296). Corroborating this, a study in HEK cells demonstrated that SLC35A1 interacts with ST3Gal-IV and that this interaction was differentially affected by CDG-causing variants in SLC35A1. This suggests that impaired transporter function may contribute to the observed glycosylation and platelet defects (296). Together, these findings support a mechanism where defective SLC35A1 lowers platelet sialylation, causing enhanced platelet clearance, thereby explaining thrombocytopenia symptoms in SLC35A1-CDG patients.

### Concluding remarks: Sia metabolism and platelets

Thrombocytopenia is a shared hallmark of patients with defects in *GNE*, *NANS*, and *SLC35A1*, with common features including giant platelets, elevated immature platelet fractions, and increased platelet clearance rather than impaired production. Although the link between platelet hyposialylation and increased clearance has been convincingly described, several aspects are still poorly understood. Interestingly, a defect in any of the Sia metabolism genes does not guarantee that thrombocytopenia will develop, since patients without these symptoms have also been reported. In addition, important differences exist in disease severity, with thrombocytopenia in GNE- and SLC35A1-deficient patients resulting in severe bleeding, while thrombocytopenia in NANS-CDG patients is often milder with more stable and higher residual platelet counts. Remarkably, GNE thrombocytopenia manifests early in childhood, while the first symptoms of GNE myopathy do not develop until adulthood. This may suggest a distinct role of the two GNE enzyme activities in platelets and muscle, differently influenced by developmental and aging processes. To gain insight into these mechanisms, additional studies are needed to reveal the cell type-specific expression of GNE isoforms and cell type-specific metabolic consequences. While macrothrombocytopenia appears specific to GNE and SLC35A1-CDG patients, other CDGs more commonly present mild thrombocytopenia without enlarged platelets (261). The exact mechanisms linking altered glycosylation to platelet lifespan and morphology also remain poorly understood. For example, the role of GPIbα surface expression and the contribution of α2,3- *versus* α2,6-sialylation defects differ across studies. Together, these rare disorders highlight sialylation as an essential regulator of platelet biology.

## Conclusion and future perspective

**Figure 2:**
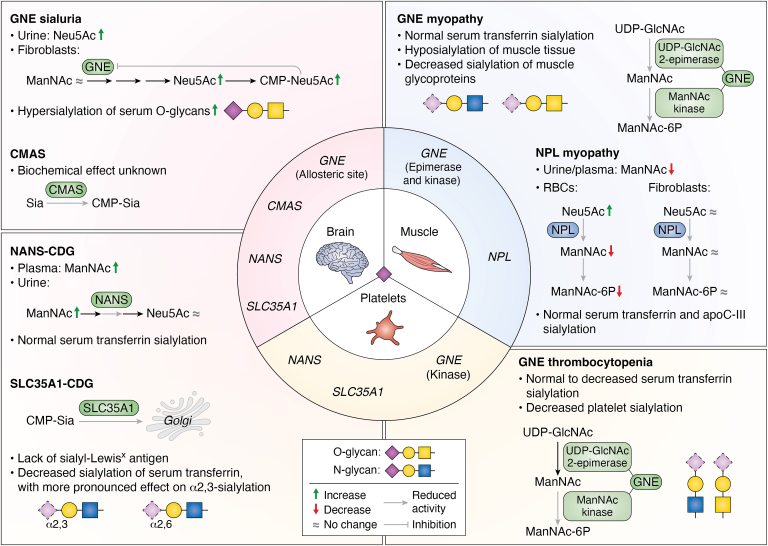
**Overview of genetic defects associated to sialic acid metabolism, grouped according to the affected tissue.** The biochemical consequences on metabolic and glycosylation level are summarized per disease. Defects in *NANS* and *SLC35A1* may affect both the brain and platelets, highlighted by the multi-colored box, while defects in *GNE* results in three distinct phenotypes. Symbols have been used to indicate changes of metabolite levels in the different diseases: ↑ (*green*) indicates an increase, ↓ (*red*) indicates a decrease, and ≈ indicates no change of the indicated metabolite level. The flat-ended line (ͱ) indicates inhibition of enzyme activity. The *gray* color of *arrows*, and flat-ended lines indicate reduced activity. RBCs, red blood cells; GNE, UDP-*N*-acetylglucosamine 2-epimerase/*N*-acetylmannosamine kinase; CMP, cytidine 5′-monophosphate; Neu5Ac, *N*-acetylneuraminic acid; CMAS, cytidine monophosphate *N*-acetylneuraminic acid synthetase; *NPL*, *N*-acetylneuraminate lyase; *NANS*, *N*-acetylneuraminate synthase; Sia, sialic acid; *SLC35A1*, solute carrier family 35 member A1; ManNAc, *N*-acetylmannosamine; ManNAc-6P, *N*-acetylmannosamine 6-phosphate; CMP, cytidine 5′-monophosphate.

Based on the comparative analysis above of different gene defects in Sia metabolism, a picture emerges of unresolved cell- and tissue-specific mechanisms explaining the tissue-restricted clinical symptoms. Sia metabolism and function appear to be especially important for the development and function of brain, muscle, and platelets; however, each genetic defect results differentially in (a combination of) symptoms related to these tissues (Fig. 2). For diseases affecting platelets, reduced sialylation has convincingly been linked to platelet lifespan and thrombocytopenia. However, the differential presence of unexplained brain and muscle symptoms in the Sia gene defects questions whether reduced sialylation is a direct cause of symptoms in other organs. This is partly related to a lack of proper (tissue-specific) model systems and the limited biochemical data on the consequences of Sia biosynthesis defects on downstream metabolic pathways and on glycoconjugate sialylation. As a result, questions regarding the overlapping and differential findings among these three tissues and how the biochemical mechanisms influence different clinical outcomes remain unanswered. Several advances are needed to address these questions and improve our understanding of Sia metabolism as a whole for development of mechanism-based therapies in human diseases with disruptions in this process.

With respect to the biochemical effect of disturbed Sia metabolism on glycosylation, a first step is the diagnostic assessment of serum transferrin N-glycosylation and apoC-III mucin O-glycosylation. While sialylation was clearly decreased for SLC35A1-CDG, normal results were found for NANS-CDG, NPL myopathy, and GNE myopathy, variable results were found for GNE thrombocytopenia, and hypersialylation was found for apoC-III O-glycosylation in GNE sialuria. While these assays mainly reflect the sialylation status of liver-derived proteins, several studies have investigated the sialylation status of affected tissues *via* lectin staining of platelets (GNE thrombocytopenia) or muscle tissue (GNE myopathy). While lectin staining provides an indication of reduced sialylation, it lacks detailed information on linkage specific sialylation or sialylation of specific glycoconjugates (lipids, N- and O-glycans, polySia). This may be highly relevant as SLC35A1-CDG for example has been associated with a specific decrease of α2,3-sialylated N-glycans by mass spectrometry (296), an effect missed by other techniques. In addition, incorporation of other forms of Sias, such as Neu5Gc and KDN, will be missed, while mass spectrometric analyses have shown incorporation of KDN in human glycans (39, 40), and differences in Neu5Gc sialylation were detected in NPL mutant mice (248). Analogous to the known differential effect of reduced UDP-GlcNAc levels on the branching of N-glycans (297), it is expected that higher or lower levels of CMP-Neu5Ac will have different effects on the activity of the large family of sialyltransferases respectively, which may link to specific clinical symptoms. Advances and application of glycomics methods to study linkage- and branch-specific sialylation on individual proteins and lipid species are needed to address this issue.

In addition to glycosylation defects, metabolic consequences directly resulting from the gene defects are important. Both NPL myopathy and GNE sialuria are for example characterised by strongly elevated levels of Neu5Ac in urine, while lowered levels in urine of Sia biosynthesis defects have not been noted. Levels of Neu5Ac were also increased in NPL erythrocytes; however, no data has been reported for other defects or for other blood cell types that are affected. Analyses of other metabolites in the Sia pathway have revealed elevated ManNAc in urine and plasma of NANS-CDG, decreased ManNAc and ManNAc 6-phosphate in NPL red blood cells, but not in fibroblasts, while CMP-Neu5Ac levels were elevated in sialuria fibroblasts. It is of interest to systematically investigate the metabolic consequences for all known genetic diseases related to Sia metabolism, including different affected and unaffected tissues and cell types. Advances are needed for easily accessible methods to quantify all sugar metabolism intermediates. Such analyses should include a distinction among the three Sia types known in humans: Neu5Ac, Neu5Gc, and KDN. In studies of the Sia pathway, analysis of intermediates in NANP KO cells has shown an accumulation of Neu5Ac 9-phosphate as expected; however, detection of normal levels of Neu5Ac and CMP-Neu5Ac led to the hypothesis of the existence of another phosphatase with similar function as NANP (44). Similarly, the exact roles of renin-binding protein and NAGK in Sia biosynthesis and recycling are unclear, and detailed metabolic analysis, including metabolic flux analysis, in appropriate model systems will be instrumental to elucidate metabolic mechanisms.

To answer many of the questions above, model systems should accurately reflect the human disease situation. Current model systems are often unable to fully replicate the complex disease phenotypes observed in patients. While mouse models capture key features of the NPL and SLC35A1 deficiencies relatively well, available models still lack accurate representation of other diseases, especially for GNE myopathy. Translation of findings from animal models to the human situation remains challenging, especially due to significant differences in Sia metabolism between humans and animal models. A major difference is the lack of *de novo* synthesis of Neu5Gc in humans, while this is abundant in most mouse tissues (30). In addition, mice carry a variant in NANS, resulting in a decreased affinity for mannose 6-phosphate (77). These key metabolic differences influence the distribution of the three major Sia types, the possibility of compensatory mechanisms, and the differences in potential toxicities of accumulating Sia forms. These complications also apply to studies using the mouse C2C12 myoblast cell line, which has been widely used in the field of skeletal muscle research, for example for modeling GNE myopathy. Another important lesson from studies on human genetic diseases is the factor of residual enzyme activity compared to the use of full KO models. While complete KO of genes in Sia metabolism will fully deplete Sia and CMP-Sia for sialylation (44), residual enzyme activity such as for NANS-CDG will allow considerable sialylation to occur. This is different from the KO brain organoid model that has been used to model NANS-CDG and may therefore miss essential subtleties in the biochemical mechanisms underlying the pathophysiology (100). It could well be that advanced organ-on-a-chip models, originating from patient-derived iPSCs or derived from iPSCs with introduced patient mutations, will fill this gap.

In addition to using model systems, a first step to gain information on potentially novel biochemical mechanisms is by comparison with other genetic defects. As most CDGs are complex clinical conditions affecting multiple organs, they result in a large number of different symptoms likely linked to the many different biological pathways involved. However, some CDGs show great similarities in their clinical spectra, and this may lead us to novel links between pathways. For GNE myopathy, similar phenotypes of inclusion bodies in muscle biopsies were found in defects in autophagy genes, such as the *SQSTM1* gene encoding for p62. For NANS-CDG, the specific type of skeletal dysplasia shows resemblance with phosphoglucomutase 3-CDG (OMIM#615816) (298). Understanding the underlying disease mechanisms of numerous CDGs can therefore contribute to solving the puzzle for other CDGs.

Mechanism-based interventions are usually very effective for treatment of metabolic diseases in case of a causal relation with clinical symptoms. Based on the hypothesis of reduced Sia production and sialylation in Sia biosynthesis defects, oral supplementation trials have been executed with Neu5Ac in GNE myopathy and NANS-CDG, unfortunately without clear clinical benefits. In view of the very short half-life of Neu5Ac, an extended release formulation (aceneuramic acid) was investigated for GNE myopathy and a trial was initiated for GNE thrombocytopenia (NCT02845609). Results were not clearly beneficial for GNE myopathy and thus far unreported for GNE thrombocytopenia. It is important to note that Neu5Ac supplementation will likely not work for biosynthesis defects beyond the Sia salvage pathway (CMAS and SLC35A1), although high dietary fucose levels were shown to be effective in rescuing childhood immune problems in SLC35C1-CDG (104). In addition, Neu5Ac supplementation will not work for GNE sialuria and NPL myopathy. ManNAc as precursor for Sia biosynthesis is currently in phase 2 clinical trials for GNE myopathy and has shown positive effects to rescue muscle symptoms in a preclinical mouse model of NPL myopathy and could be considered for GNE thrombocytopenia. Overall, the mechanisms of *in vivo* uptake and metabolism of dietary monosaccharides, such as ManNAc and Neu5Ac, should be investigated in more detail, as well as additional pharmacological formulations to improve delivery to specific tissues. Finally, in view of the many open questions on the biochemical mechanisms as discussed in this review, mechanism-agnostic approaches such as gene therapy are increasingly being studied to target defects in Sia metabolism (219, 220).

This review has extensively summarized the biosynthetic and genetic pathways related to Sia metabolism and compared the consequences of its genetic defects for brain, skeletal muscle, and platelets. Among these three tissues, its crucial role in platelets seems to be the most resolved, while many open questions still remain on the underlying mechanisms for skeletal muscle and brain. Future approaches should include systematic studies of metabolites and sialylation in both affected and unaffected tissues from patients, and in derived mutant model systems, to deeply understand the tissue-specific mechanisms in Sia metabolism and to develop novel targeted therapeutic strategies.

## Conflict of interest

The authors declare that they have no conflicts of interest with the contents of this article.
